# Prediction and Interpretability Study of the Glass Transition Temperature of Polyimide Based on Machine Learning and Molecular Dynamics Simulations

**DOI:** 10.3390/polym17152083

**Published:** 2025-07-30

**Authors:** Wenjia Huo, Boyang Liang, Xiang Wu, Zhenchang Zhang, Weichao Zhou, Haihong Wang, Xupeng Ran, Yaoyao Bai, Rongrong Zheng

**Affiliations:** School of Petrochemical Engineering, Shenyang University of Technology, Liaoyang 111003, China; huowenjia2001@126.com (W.H.); leno0120@126.com (B.L.); wux0114@163.com (X.W.); zzc10007@163.com (Z.Z.); 13062005507@163.com (W.Z.); whhq1717@163.com (H.W.); 18635179037@163.com (X.R.); baiyaoyao2017@163.com (Y.B.)

**Keywords:** polyimide, machine learning, glass transition temperature, molecular dynamics simulation

## Abstract

The utilization of machine learning (ML) has brought more opportunities for the discovery of high-performance materials with specific properties to replace traditional engineering materials. The glass transition temperature (T_g_) is a crucial characteristic of polyimide (PI). But small datasets can only partially reveal structural information and decrease the ability of the models to learn from the observed data. In this investigation, a dataset comprising 1261 PIs was assembled. A quantitative structure–property relationship targeting T_g_ was constructed using nine regression algorithms, with the Categorical Boosting demonstrating the highest accuracy, achieving a coefficient of determination of 0.895 for the test set. SHapley Additive exPlanations analysis identified the NumRotatableBonds descriptor had a significantly negative impact on T_g_. Finally, all-atom molecular dynamics (MD) simulations calculated eight PI structures to verify the accuracy of the prediction model. The ML prediction was consistent with the MD simulation, with the lowest prediction deviation of approximately 6.75%, but the time and resource consumption were tremendously reduced. These findings emphasize the significance of utilizing extensive datasets for model training. This available and interpretable ML framework provides impressive acceleration over the MD simulation and serves as a reference for the structural design of PI with the desired T_g_ in the future.

## 1. Introduction

Polyimide (PI), one of the promising engineering plastics of the 21st century, has excellent chemical stability, outstanding heat resistance, low dielectric constant, and remarkable mechanical properties [1,2,3]. It has been widely used in aerospace, microelectronics, flexible display technology, photovoltaic cells, and automotive industries [4,5,6]. Its applications covered various types such as films, resins, fibers, and adhesives, earning it the title of “golden film” [7,8,9]. In 1955, DuPont synthesized an aromatic PI and applied for the first patent on PI [10]. In 1969, Frazer et al. [11] synthesized a PI with excellent thermal stability by combining pyromellitic dianhydride and 4,4′-oxydianiline, achieving the commercialization of PI. PI can be polymerized from polycondensation reactions between dianhydride and diamine or diisocyanate monomers. The unique molecular structural characteristics, such as the conjugation of the nitrogen five-membered ring and six-membered ring, the formation of intramolecular and intermolecular charge transfer complexes, make PI have excellent properties such as high T_g_. T_g_ is a critical physical parameter for polymers, marking the temperature threshold at which cooperative segmental motion initiates, transforming the material from a rigid glassy state to a flexible rubbery state [12]. High T_g_ PI has been widely utilized in high-temperature resistant composites for spacecraft and satellites, optoelectronic devices, flexible displays, and sealing materials under high-temperature and high-pressure environments [5,13]. Flexible display technology requires materials with high T_g_ and excellent light transmittance. High T_g_ PI can maintain good transmittance even at high temperatures [14]. Therefore, the design of high T_g_ PI has become a hot topic in the field of materials science. Currently, conventional methods for measuring T_g_ rely on experimental testing, which suffer from high costs and long cycle times and have difficulty in accelerating material design and effectively predicting the performance of novel PIs. The latent PI structures can reach millions, making it impractical to navigate their structure–property landscapes under conventional experimental screening. Traditional simulation approaches, such as density functional theory [15], finite element analysis [16], and molecular dynamic (MD) simulation [17], can mitigate these obstacles, but they still exhibit certain limitations, including time-intensive computations and a substantial reliance on expert judgment in selecting simulation parameters. In summary, the testing cycle and complex calculations have constrained the pace of novel material development. Therefore, there is an urgent need to develop a more efficient approach for identifying high-performance PI materials.

The T_g_ of PI is highly dependent on the chemical structure, although the processing conditions cause T_g_ to fluctuate within a certain range. From an experimental aspect, the strategic design of molecular structures plays a pivotal role in achieving the targeted high T_g_. In recent years, with the rapid development of data-driven technologies, machine learning (ML) has emerged as a novel approach in materials development, which focuses on utilizing data and specific algorithms to simulate human learning processes [18]. It transforms traditional trial-and-error experimental methods into theoretical prediction-guided experiments [19,20]. ML has become the frontier direction and hotspot of materials research in many fields, including energy materials [21], resin materials [22], high-entropy materials [23], membrane materials [24], polymer dielectrics [25], solid-state materials [26], organic light-emitting diode materials [27], and photovoltaics materials [28]. Compared to other polymers, PIs have a larger structure-property database due to the diversity of the dianhydride and diamine or diisocyanate structures, and their combinations. This serves as a crucial foundation for establishing quantitative structure–property relationships (QSPRs) using ML approaches to accelerate molecular design [29,30]. ML algorithms have been widely applied to predict the dielectric, thermal, mechanical, optical, and solubility properties of PI [31,32,33]. Zhang et al. [34] developed a rapid and accurate ML approach to design PI candidates with the desired T_g_ value. They collected a dataset of 878 PIs and sequentially compared six ML regression methods, and the Artificial Neural Network (ANN)-based model gave the most accurate results with the root mean square error (RMSE) of 11 K. This accuracy has reached the level of the traditional molecular simulation. Zhang et al. [35] focused on four major categories of properties of PI and trained 176 models for 11 predictions of properties. High-performance PIs for three advanced fields were finally selected through high-throughput screening of nearly 7.6 million PIs based on the trained model. Wen et al. [36] explored three different approaches to either compute T_g_ for PIs via all-atom MD simulation or predict T_g_ via a mathematical model generated using ML algorithms to analyze existing data collected from literature. The best prediction model achieved a correlation coefficient value of 0.83 and an average error of 17.98 K. The prediction model derived with ML algorithms could be used to estimate T_g_, even for PI yet to be synthesized experimentally. Volgin et al. [37] developed a graph convolutional neural network (GCNN) to predict the T_g_ as an example of the fundamental property of polymers. To train the GCNN, they proposed a “transfer learning” approach with an enormous “synthetic” dataset for pretraining and a small experimental dataset for its fine-tuning. GCNN achieved a mean absolute error (MAE) of 22.5 K and a coefficient of determination (R^2^) of 0.62. Luo et al. [38] combined literature data, ML, and MD simulation to identify key factors influencing the stability of PI structures and screen for high-stability candidates. This approach offered valuable insights for the development of high-stability polymers. Qiu et al. [39,40] utilized a graph neural network to build a regression model for predicting T_g_ of PI and a classification model for two-step ensemble molecular screening. A total of 372 data points were obtained for modeling. The model offered a visual interpretation of the effect of functional group variations on T_g_. A total of 110 alternative PI structures with T_g_ exceeding 400 °C were derived, which can be used by scientists for structure filtering. Tao et al. [41] established multiple ML models for the thermal and mechanical properties of PI based on their experimentally reported values. Their study demonstrated an efficient way to expedite the discovery of novel polymers using ML prediction and MD validation. However, in the previous studies mentioned above, small samples of data affected the accuracy and interpretability of the models.

**Figure 1:**
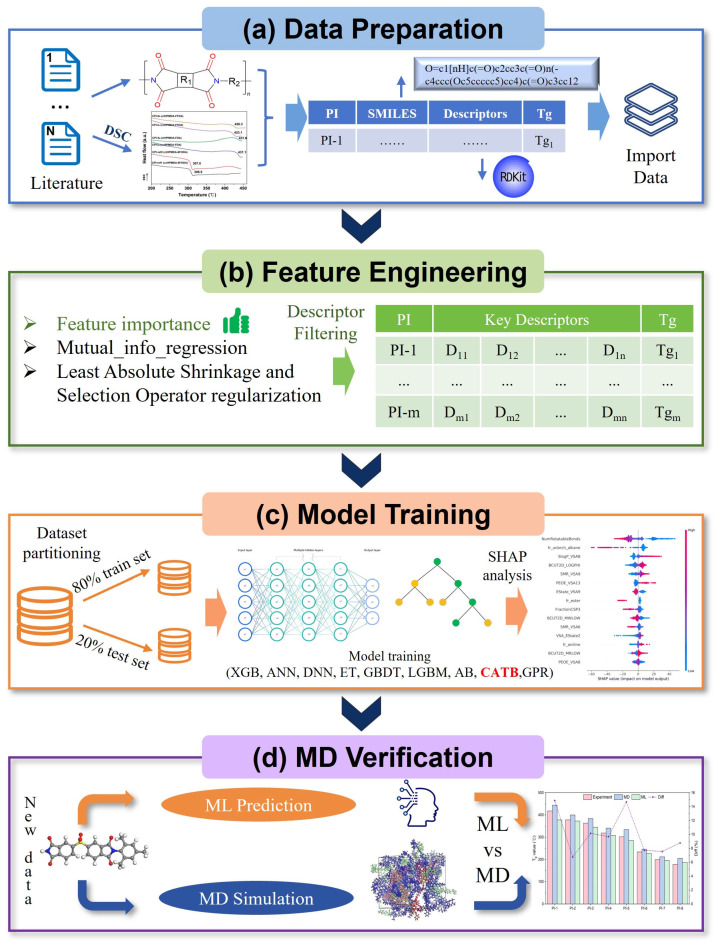
The main flowchart of machine learning in this study, includes (**a**) data preparation, (**b**) feature engineering, (**c**) model training, and (**d**) MD verification.

In this context, ML techniques driven by large-scale traditional experimental databases have brought new developments to the fields of property prediction and polymer design. Exhausting all possibilities using data-based ML prediction before experimental synthesis successfully allows the exploration of the whole design space. Moreover, the quality of the training data significantly impacts the efficiency and accuracy of ML tasks. When the number of training samples is limited, the model’s ability to learn from the observed data sharply declines, resulting in poor predictive performance [42]. For this reason, this study collected a large number of PIs, screened and standardized these structures, and finally obtained 1261 data points, thereby establishing a large-scale and comprehensive dataset. These chemical structures were first converted into Simplified Molecular Input Line Entry System (SMILES) notation [43]. From the unique SMILES string of each PI, 210 descriptors were extracted as feature representations using the RDKit package (2023.3.2) [44]. Three feature selection methods were compared to further simplify the descriptor set. The selected descriptors were ultimately used as model inputs. Subsequently, we constructed the T_g_-QSPR model using nine popular ML algorithms, all of which demonstrated high accuracy and stability. Among them, the Categorical Boosting (CATB) model demonstrated the highest prediction accuracy, achieving an R^2^ of 0.895 for the test set, MAE of 18.58 °C, and RMSE of 23.06 °C. Additionally, with the assistance of SHapley Additive exPlanations (SHAP), the contribution of each descriptor to T_g_ was revealed to enhance the interpretability of models, thereby assisting in material design. Finally, eight selected PIs with different T_g_ values not included in the original dataset, with the highest T_g_ exceeding 400 °C, were calculated through all-atom MD simulation to validate the ML prediction results. The lowest prediction deviation of the CATB model was found to be 6.75% by comparison of the MD-calculated and ML-predicted values. Next, ML prediction and MD simulation were compared with experimental measurements from published literature, highlighting the performance of the ML model. Further investigation into the interpretability of the models, along with MD validation, significantly enhanced the practical utility of the models. Based on the big material database, our study demonstrates the feasibility of using the ML-QSPR method to design PIs with desired T_g_. This approach can be extended to evaluate the relationship between PI structures and other properties, and also provide an innovative strategy for guiding the performance optimization and rational design of other novel materials. The next section details the entire methodology implemented in this work, which addresses both the data-driven strategy and the molecular modeling aspects. The workflow of this study is illustrated in Figure 1.

## 2. Materials and Methods

### 2.1. Database and Feature Selection

More than 1500 molecular structures and T_g_ values of PI were collected from published literature to construct the structure–property relationship models. T_g_ data comprised both experimental and theoretical values. Since T_g_ can be determined through various measurement methods, including expandometer, thermo-mechanical analysis (TMA), dynamic mechanical analysis (DMA), differential scanning calorimetry (DSC), etc., differences among these techniques may introduce errors in T_g_ values. To minimize the measurement error, this study uniformly employed DSC-measured T_g_ values. Furthermore, considering the influence of different testing methods, conditions, and synthesis approaches on the T_g_ values of PI, the average value with a sufficiently small standard deviation was adopted as the representative when encountering different T_g_ values for the same molecular structure. In this study, the repeating unit of each polymer was used to represent the overall molecular structure. Each repeating unit of PI contained two unsaturated single bonds at both ends. To address this, hydrogen atoms were added to cap the ends, transforming the repeating unit into a complete molecular structure. Subsequently, the repeating unit structures were drawn using ChemDraw 20.0 and then individually converted into the SMILES expression using the built-in SMILES encoding module of ChemDraw (Edit > Copy As > SMILES), which is a simplified molecular linear input specification that employs ASCII characters to describe polymer structures [45]. SMILES uses atomic symbols, bond types (single, double, or triple), and parentheses to represent branches, encoding molecular topology in a linear format to explain the connections between atoms and simplify the dimensions of molecular information into a single string. To avoid different SMILES encoding forms for the same molecular structure, the MolStandardize module of the open-source cheminformatics toolkit RDKit package was used to standardize the converted SMILES characters in a uniform manner. After standardizing and screening all structures, a total of 1261 structural–property data were compiled into the T_g_ dataset. Based on SMILES, 210 molecular descriptors suitable for ML were calculated using the RDKit package embedded in Python 3.7.3. Molecular descriptors, as a popular form of feature representation, encompass general descriptors, molecular charges, bond positions, geometric descriptors, fragment descriptors, etc. Compared to more advanced representations such as graph-based or embedding-based descriptors (e.g., molecular fingerprints or graph neural networks), these descriptors can capture comprehensive information such as molecule size, shape, types of chemical bonds, and substituent groups to quantify structural and physicochemical properties of the compounds and provide direct, chemically intuitive mappings to properties [46]. Although the experimental process caused T_g_ to fluctuate within a certain range, specific structural features of molecules highly affect the arrangement of PI molecules, intermolecular interactions, chain mobility, and ultimately influence their T_g_. The calculated molecular descriptors served as key quantitative indicators for the structure–property relationships, enabling the transformation of molecular structures into machine-interpretable feature spaces. However, excessive input features may lead to overfitting (excessive adaptation to the training data) during model training, consequently increasing the model complexity and reducing prediction accuracy. To extract key descriptors for constructing prediction models and reduce data dimensionality and computation time, it is necessary to evaluate the original descriptors and identify those with a significant impact on model performance. Thereby, all descriptor columns with all zero values in the calculation were first eliminated. In the second step, three methods—feature importance [47], mutual_info_regression [48], and Least Absolute Shrinkage and Selection Operator (LASSO) regularization [49]—were employed to further screen key descriptors. The selected descriptors were considered as inputs for constructing the T_g_ prediction models.

### 2.2. Machine Learning Methods

Based on the diversity of the dataset, we tried various algorithms to predict T_g_ of PI. Comparing many algorithms helped find the optimal model to improve the accuracy of prediction results and reduce errors. Therefore, nine popular ML algorithms, including eXtreme Gradient Boosting (XGB) [50], ANN [51], Deep Neural Network (DNN) [52], Extra Trees (ET) [53], Gradient Boosting Decision Tree (GBDT) [54], Light Gradient Boosting Machine (LGBM) [55], AdaBoost regression (AB) [56], CATB [57], and Gaussian Process Regression (GPR) [58] algorithms, were employed to establish the structure–property relationship of PI and explore the impact of different algorithms on prediction accuracy. In other words, we comprehensively compared nine ML models and three feature selection methods for the T_g_ prediction. The dataset was split into a training set consisting of 1009 PIs (80%) and a test set containing 252 PIs (20%). The test set was excluded from the model construction process. During the model training, the selected descriptors were utilized as inputs to the model, and the predicted T_g_ values served as outputs. To further improve the accuracy of model predictions, the GridSearchCV method was employed to search for the optimal combination of hyperparameters for each regression learning model instead of using default parameters. Subsequently, the 10-fold cross-validation was conducted to test the stability of the models. The error between the actual and predicted values served as a metric for evaluating the prediction performance of different models. Herein, we introduced three key statistical parameters, which were calculated according to Equations (1)–(3), including R^2^, MAE, and RMSE, to assess the goodness of fit and generalization ability of training models to find the optimal model [59]. The R^2^ value, which is a unitless metric, quantifies the proportion of variance in the dependent variable that is explained by the model. MAE measures the average magnitude of errors by calculating the absolute differences between predicted and actual values. RMSE, in contrast, quantifies the standard deviation of prediction errors by penalizing larger discrepancies quadratically. RMSE and MAE on the training and test sets demonstrate a certain degree of consistency.

(1) $$R^{2}=1-\frac{\sum_{i=1}^{N}{\left(y_{i}-{\hat{y}}_{i}\right)}^{2}}{\sum_{i=1}^{N}{\left(y_{i}-\bar{y}\right)}^{2}}$$

(2) $$MAE=\frac{1}{N}\sum_{i=1}^{N}\left|y_{i}-{\hat{y}}_{i}\right|$$

(3) $$RMSE=\sqrt{\frac{1}{N}\sum_{i=1}^{N}{\left(y_{i}-{\hat{y}}_{i}\right)}^{2}}$$

### 2.3. Model Interpretation and Validation

After establishing the ML regression models mentioned above for PI, we needed a method to explain the key influencing factors to guide the design of future materials and thus improve the interpretability of the models. The SHAP method is one of the most powerful ML interpretable analytical tools available today, and has been used in many research [60]. SHAP provides a mathematically rigorous framework for interpreting complex ML models by quantifying feature importance through cooperative game theory principles. By leveraging the Shapley values, the marginal contribution of each feature to the prediction is calculated, which in turn quantifies the impact of each input variable on the final prediction results. Through summary visualizations and local explanation plots, SHAP simultaneously delivers both global interpretability (whole-model feature ranking) and local explainability (individual prediction breakdowns), enabling the identification of critical molecular descriptors driving target properties. This dual-scale insight is particularly valuable for confirming the structure–property relationships and discovering feature interactions in materials science. Thereby, in this study, according to SHAP analysis, the significant contribution of each physicochemical descriptor to the model output results can be obtained.

**Figure 2:**
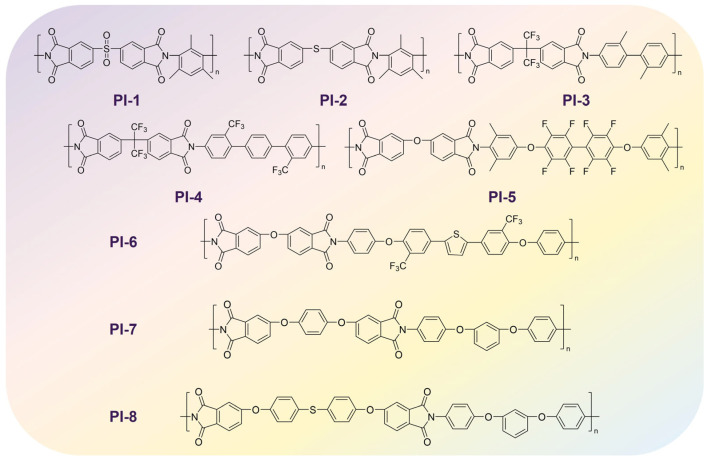
Structures of PIs (PI-1–PI-8) for validating the accuracy of the ML prediction model.

In order to validate the accuracy of the model, we identified eight types of PI films with different thermal performance that do not exist in the database, with the highest T_g_ exceeding 400 °C. PI-1 to PI-8 were obtained from different recently published literature sources [61,62,63,64,65,66,67] and have been shown to have been synthesized, exhibiting significant and diverse structural variations. Figure 2 illustrates the molecular structures of PIs. They had rich structural features and significantly different T_g_ values, ranging from 100 to 400 °C. Subsequently, an all-atom MD simulation was performed to simulate and calculate their T_g_. MD simulation is a computer-based method of deep computation, which computationally models atomic-scale behavior by solving Newton’s equations of motion under empirically validated force fields. This approach tracks femtosecond-scale trajectories of atoms within specified thermodynamic ensembles (NPT/NVT) [68], captures fundamental processes as molecular diffusion and conformational changes, and obtains the corresponding macroscopic properties from the microscopic properties. In addition, MD allows a careful evaluation of the dependence of T_g_ on the structural factors like chain rigidity, intermolecular interactions, and fractional free volume. As the temperature is raised and approaches the T_g_, polymer segments gain sufficient thermal energy to initiate large-scale cooperative motions. Around this temperature, the free volume begins to increase at a significantly faster rate, enhancing molecular mobility and flexibility. This marks the transition from the rigid glassy state to the flexible rubbery state. The increase in free volume and chain mobility profoundly impacts the material’s properties. Consequently, the specific volume exhibits a characteristic change in its temperature dependence. Plotting specific volume versus temperature reveals a distinct change in slope. In this study, the T_g_ of the PI was determined using this specific volume versus temperature method. MD simulation was performed by using Materials Studio 2020 modeling software. In MD, the T_g_ can be calculated via annealing dynamics. Initially, the Sketch module was used to draw the structural monomer of PI. PI chains containing 15 repeating units were constructed with random torsion angles using the Build Polymers module. Amorphous Cell (AC) models representing the aggregated state were constructed for each system using the AC module. Each AC contained 10 molecular chains. In order to approach the actual situation, the initial density of each simulation cell was set to 1.4 g/cm^3^ at 298 K. Twenty distinct AC structures were generated, each of which underwent full geometry optimization, and the lowest-energy AC structure was selected for the subsequent dynamic optimization simulations (the lower the energy, the more stable), followed by geometry structure optimization using the Forcite module. Subsequently, MD simulation replicated the physical cooling process of polymers by cooling from 900 K to 300 K in decrements of 10 K to obtain the average density of each system after stabilization at different temperatures using the Forcite module and calculate specific volume. The time step was 1 fs, and the total simulation time was 500 ps under NPT ensemble. The cooling rate was 20 K/ns. MD simulation consistently employed the COMPASS III force field throughout. Asymptotically, the specific volume varies linearly with respect to temperature in both the glassy and the rubbery regimes, however with different slopes. In practice, two linear functions can be fitted to the low-temperature and high-temperature domains, respectively. As the system temperature gradually decreased, segmental mobility became kinetically arrested, manifesting as a slope discontinuity in temperature-dependent properties. The specific volume was tracked at each temperature step during NPT ensemble simulations. Plots of specific volume versus temperature exhibited a significant characteristic change in slope when amorphous systems changed from rubbery to glassy state [69], with T_g_ identified as the intersection point of two linear regressions fitted to the low-temperature (glassy) and high-temperature (rubbery) regimes of the specific volume–temperature curve. This computational method captured the temperature-dependent volumetric changes associated with the glass transition observed during cooling, while correcting for cooling-rate errors.

## 3. Results

### 3.1. Data Collection and Feature Extraction

**Figure 3:**
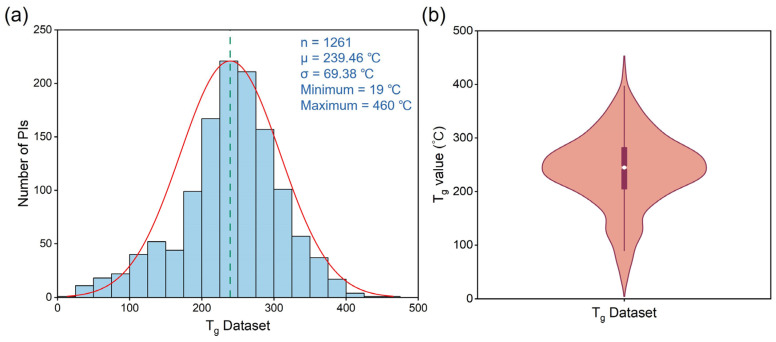
T_g_ distribution of PIs collected from the literature. (**a**) The green dashed line shows the mean value; the red solid line shows the normal distribution curve. n is the data sample size, μ is the mean value, and σ is the standard deviation. (**b**) The violin plot shows the upper quartile, median, and lower quartile of T_g_ dataset.

In this study, the dataset used for ML models training comprised 1261 PI molecular structures, and their T_g_ values were measured by DSC. The structures were derived from approximately 240 different articles. Details of the structures and source literature can be found in Supplementary Materials (Table S1). The collected data revealed a significant diversity of PI chemical structures, encompassing a broad chemical space that included both aliphatic and aromatic PIs. T_g_ values covered a wide range, ranging from 19 °C to 460 °C. The variance was approximately 4813. The larger variance value indicated significant differences among data points, leading to a broader data distribution. The T_g_ data were most abundant between 200 °C and 300 °C, with a mean of 239.46 °C and a standard deviation of 69.38 °C, as shown in Figure 3a. The distribution of T_g_ can be approximated to a normal distribution. The upper quartile of the data distribution is 282 °C, the median is 245 °C, and the lower quartile is 205 °C (Figure 3b). Subsequently, the PI structures were converted into SMILES strings.

**Figure 4:**
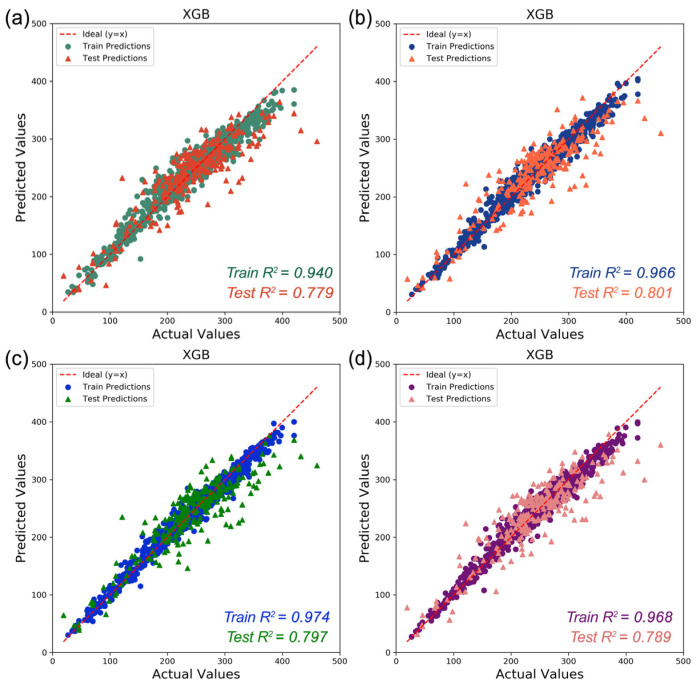
The scatter plot of the R^2^ prediction results of the XGB model when using different methods (**a**) removing zero column; (**b**) feature importance; (**c**) mutual_info_regression; (**d**) LASSO regularization) to filter descriptors.

**Figure 5:**
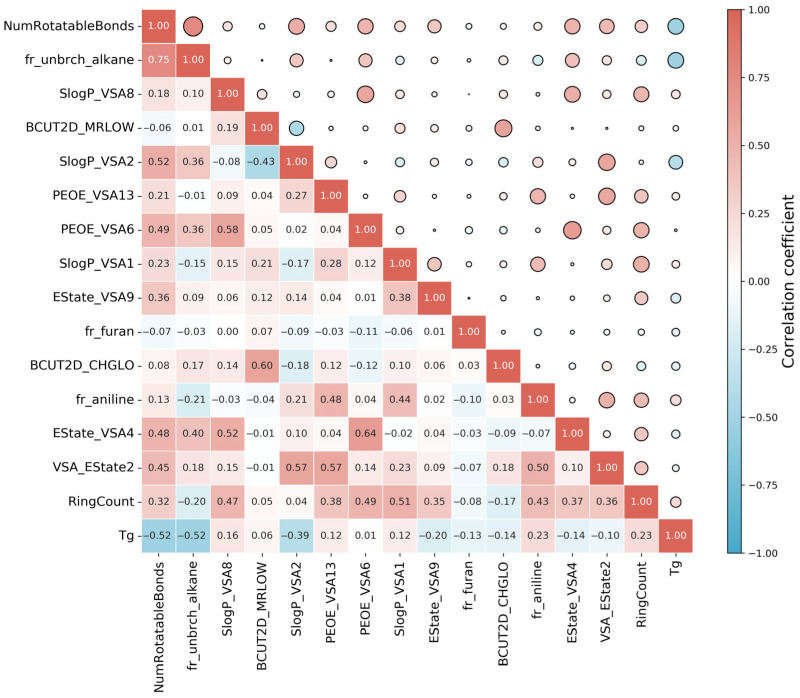
Correlation analysis between the top 15 molecular descriptors and T_g_, as well as the PCC between each descriptor. The larger the circles, the stronger the correlation between each descriptor.

The SMILES of each PI structure was converted into descriptors using RDKit, which was a Python toolkit that calculated 210 2D molecular descriptors, as illustrated in Table S2. These descriptors responded to the physicochemical, topological, and charged properties of the polymers as well as the intermolecular forces. Not all these features played important roles in affecting the T_g_. To simplify the model inputs, all descriptor columns with all zeroes in the calculation were first eliminated, resulting in the 175 most valuable descriptors. Here, we employed three feature selection methods to further refine the features, including feature importance, mutual_info_regression, and LASSO regularization. The feature importance method employed the Random Forest (RF) algorithm to calculate feature importance scores and ranked them accordingly. Descriptors with importance scores above the overall average were first retained. To eliminate the highly similar and overlapping descriptors, the Pearson correlation coefficient (PCC) [70] was calculated to analyze the correlation coefficients of the retained descriptors. The smaller the value, the lower the correlation. Low correlation indicated that the molecular features provided relatively independent information, with less redundancy among the features. Therefore, for strongly correlated descriptors (PCC > 0.85), we prioritized retaining those with higher importance scores to address multicollinearity issues while ensuring the removal of redundant variables without compromising critical structural information. Mutual_info_regression is an information-theoretic feature selection method. Rooted in information theory, mutual information (MI) can be used to quantify the dependence between features and the target variable. It is particularly effective for analyzing high-dimensional datasets. Features are ranked by their normalized MI scores, with values close to 1 indicating strong information related to T_g_. Based on the MI, a threshold was applied to retain descriptors that exhibited statistically significant dependencies. LASSO regularization is implemented to simultaneously perform feature selection and regularization by imposing an L1 penalty on the molecular descriptor coefficients. It can effectively shrink coefficients of non-informative features to zero by inducing sparsity while retaining chemically relevant predictors for T_g_. By adjusting the penalty factor (λ), the feature selection intensity can be controlled. If λ is 0, there will no shrinkage of any of the 175 features, and LASSO regularization becomes ordinary linear regression. A big positive value of λ indicates that the majority of the features will be removed. In this experiment, the λ values were manually adjusted and tested across a broad range (from 0.1 to 1). All screened descriptors using the above methods can be found in the Supplementary Materials. Taking XGB as an example, Figure 4 shows a scatter plot of the R^2^ prediction results using different feature screening methods. The closer the R^2^ value is to 1, the greater the model’s explanatory ability. The three key statistical parameters on the training and test sets and 10-fold cross-validation are shown in Table 1. The results showed that all feature selection methods significantly improved the prediction accuracy of the model. Among them, the R^2^ of XGB prediction model constructed based on the descriptors filtered by the feature importance method on the test dataset reached 0.801, which was better than the other two methods. All in all, the model trained for descriptor screening using feature importance for regression algorithms such as XGB had the highest R^2^, lower MAE, and RMSE. Moreover, the standard deviation of the cross-validation of the feature importance method was relatively small, indicating that the T_g_ prediction model constructed with the descriptors screened by this method had better stability. Therefore, we chose this method to filter descriptors and ended up with 43 descriptors as input. Figure 5 shows the PCC between the top 15 selected descriptors and T_g_, as well as the heat map of the PCC between each descriptor.

### 3.2. Performance Evaluation of the ML Models

**Figure 6:**
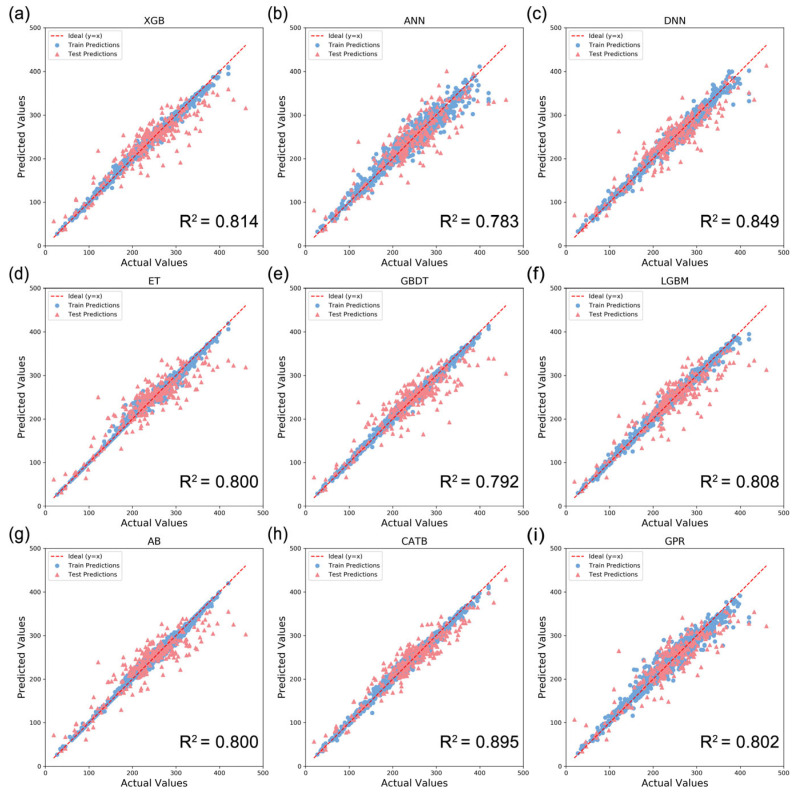
ML performance. Comparison of nine ML regression algorithms ((**a**) XGB, (**b**) ANN, (**c**) DNN, (**d**) ET, (**e**) GBDT, (**f**) LGBM, (**g**) AB, (**h**) CATB, and (**i**) GPR) to construct the T_g_-QSPR models. The blue dots represent the prediction results of the training set, and the pink dots represent the prediction results of the test set.

**Figure 7:**
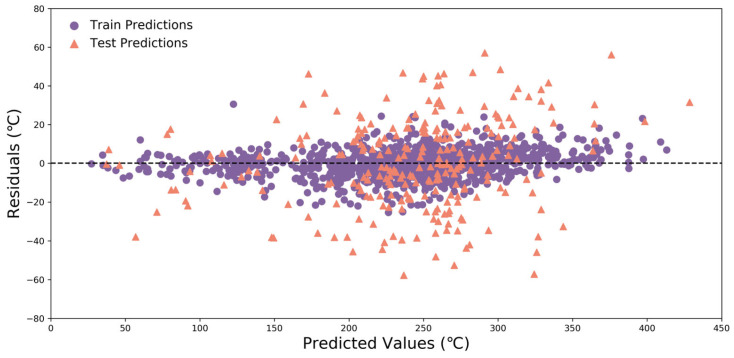
Residual diagram of CATB model. The distribution of residuals (actual–predicted values) on the training and test sets.

For T_g_ prediction, different models were established and compared to the perspectives of the feature extraction and algorithm selection. The goodness-of-fit of each model was evaluated using three statistical parameters R^2^, MAE, and RMSE. Nine popular ML algorithms, including XGB, ANN, DNN, ET, GBDT, LGBM, AB, CATB, and GPR were adopted to construct T_g_-QSPR models using the above 43 descriptors as inputs. The Supplementary Materials showed the prediction results using three feature filtering methods and nine algorithms (Figures S1–S3). All the models were trained using their default parameters. As shown in Figure 6, after hyperparameter tuning through GridSearchCV, the R^2^, MAE, and RMSE of all models had improved, highlighting the necessity of hyperparameter optimization in the model construction process. The dashed line indicated the case when the predicted value was equal to the actual value. The closer the line was to the dashed line, the smaller the error between predicted and actual values, indicating a higher accuracy. In summary, by using the CATB algorithm, the optimal T_g_ prediction model can be obtained with an accuracy of R^2^ = 0.989, MAE = 5.37 °C, and RMSE = 7.08 °C, as measured on the training set; R^2^ = 0.895, MAE = 18.58 °C, and RMSE = 23.06 °C, as measured on the test set. The training and test sets were distributed evenly on both sides of the diagonal, further corroborating its stability. Moreover, the standard deviation of 10-fold cross-validation of CATB model was smaller, which meant that the CATB prediction model had higher generalization performance and better stability (Table 2). The residual is the difference between the actual value and the predicted value. As illustrated in Figure 7, the majority of residuals were concentrated near the zero line, indicating that the model’s prediction ability was satisfactory. There was no discernible pattern or trend in the residuals. This indicated that the model’s predictions were uniform across all value ranges, and there were no systematic errors in certain specific areas. This further verified the robustness and generalization ability of the model. The distribution of the training and the test set on both sides of the zero line was similar, indicating that the model performed consistently on the training set and the test set, with no obvious overfitting or underfitting.

In this study, the majority of ML models demonstrated excellent prediction performance, with the R^2^ of greater than 0.80 on the test set, while the ANN-QSPR model and GBDT-QSPR model were not acceptable with R^2^ < 0.8. Among them, the CATB model had the strongest learning ability and the highest R^2^ values for the test set, followed by the DNN-QSPR. XGB, GBDT, LGBM, and CATB belong to gradient boosting frameworks. They are built-in learners based on Boosting algorithms, which incorporate multiple weak learners (decision trees) to enhance the learning ability of the models. XGB, GBDT, and LGBM models performed well on the training set, but their test results were much lower than the training results, exhibiting overfitting. This is because ensemble learning has more base learners, which makes the model too complex and leads to overfitting. Compared with above algorithms, CATB demonstrates relative robustness in the hyperparameter selection, automatically handling most hyperparameter adjustments with minimal impact from outliers. This makes it better handle noise and exceptional situations, effectively suppressing overfitting induced by noisy or correlated features. AB is also an ensemble method under the boosting paradigm, which combines multiple weak learners, but there are fundamental differences in optimization mechanisms and error correction strategies. AB adaptively adjusts the sample weights to prioritize misclassified samples. But outlier samples can affect the prediction accuracy. Neural network algorithms, such as ANN and DNN, can utilize layered nonlinear transformations to capture complex patterns. The foundational architecture of the neural network comprises three functionally distinct layers: the input layer, hidden layer(s), and output layer. Compared with the ANN, DNN is a multilayer neural network (≥3 hidden layers), which can employ regularization and dropout methods to reduce overfitting and improve the prediction performance. By using large neural networks in the DNN model, complex models can be trained, and better results can be obtained. In order to capture as much of the complex nonlinear relationship between polymer structure and performance as possible without overfitting the model, our DNN model contained four hidden layers and four dropouts. The number of neuron nodes within each hidden layer of the DNN model was adjusted using manual parameter tuning. However, DNN requires a larger data and computational resources. For some simple tasks, it is more sophisticated than traditional ML algorithms, resulting in performance degradation. ET is a bagging-type learner, which is a parallel ensemble algorithm composed of decision trees. The correlation between the base learners is reduced through bootstrap sampling. But this algorithm requires high data balance and is sensitive to abnormal samples, causing the unsatisfactory prediction results. GPR is a powerful non-parametric Bayesian ML technique, which is used for probabilistic regression and uncertainty estimation. It critically depends on kernel selection and hyperparameter tuning, where poor choices can lead to overfitting or underfitting, and its performance may degrade with high-dimensional inputs due to the curse of dimensionality. Composite kernels (e.g., combining linear, periodic, and radial basis functions) can enhance expressiveness for complex data structures. The optimal parameter information of different models is shown in the Note S1.

### 3.3. Interpretability Analysis of the Model Using SHAP

**Figure 8:**
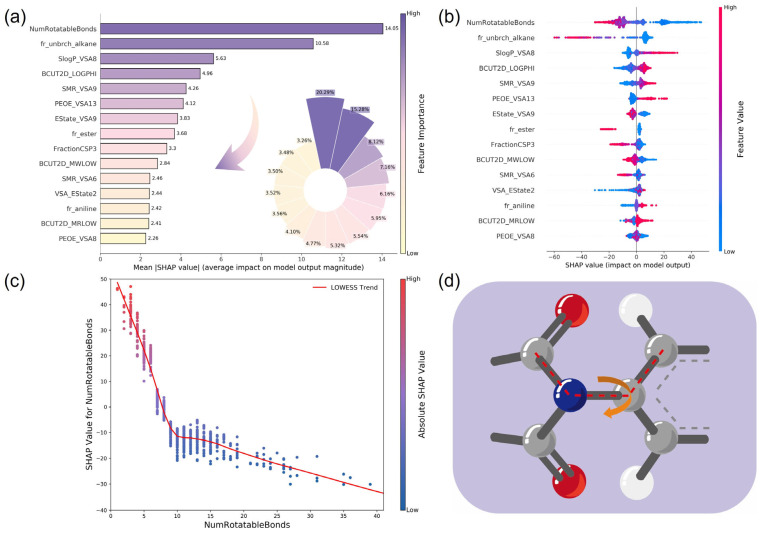
Using SHAP summary plots ((**a**) bar plot and (**b**) dot plot) to sort the impact of the top 15 most important descriptors on T_g_ from largest to smallest. (**c**) SHAP dependence plot. It visualizes that the T_g_ and NumRotatableBonds exhibit a negative correlation trend. (**d**) Chemical interpretation of the NumRotatableBonds descriptor. Arrows indicate the rotatable nature of chemical bonds.

**Figure 9:**
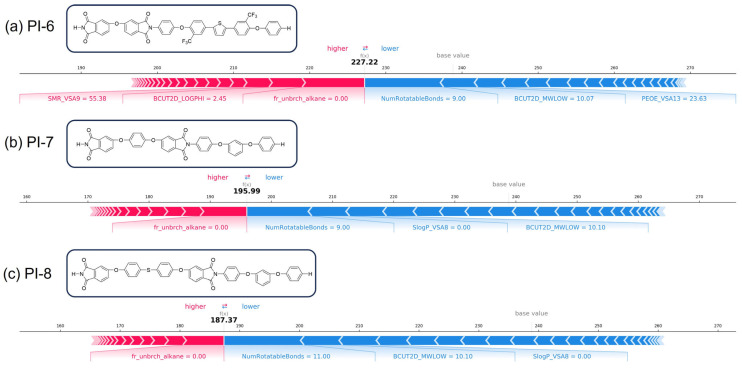
Force Plot. Descriptor impact analysis on T_g_ prediction for three representative PI structures. (**a**) PI-6, (**b**) PI-7, and (**c**) PI-8.

T_g_ is an important criterion for evaluating polymer performance in several applications, reflecting the rigidity and heat resistance of the polymer molecule. To further investigate the relationship between the structural characteristics and T_g_ of PI, we utilized the SHAP analysis to explore the chemical implications of the key selected descriptors and improve the interpretability of the CATB model. Figure 8a visualized the top 15 most important descriptors related to T_g_. The average absolute SHAP values for each descriptor were used to determine its importance. As shown in Figure 8b, each row represented a descriptor, with the *X*-axis indicating the SHAP value (positive values enhanced T_g_, negative values reduced T_g_). Each point represented a sample, with the color indicating the descriptor magnitudes (red for high, blue for low). Based on the summary plot figures that combined feature importance and feature effects, we could not only assess the magnitude of the impact of descriptors on T_g_, but also analyze the positive or negative effects of their specific values. This approach provided a better understanding of the overall patterns and allowed for the identification of outlier predictions. Figure 9 presents a force plot that visually compares the impacts of descriptors on T_g_ predictions for three distinct samples. The base value (238.83), calculated as the model’s average predicted T_g_ over the entire background dataset of 1261 PI samples, served as the initial reference point of the model. The f(x) values denoted the actual predicted T_g_ for individual samples. These predictions were calculated relative to the baseline, with descriptors enhancing the T_g_ highlighted in red and those reducing it in blue. The numerical annotations adjacent to each descriptor indicated their specific descriptor values. The feature value of a substructure can be “0”, meaning the absence of the descriptor in the molecule, but its feature importance was still a valid value indicated by the length of the arrow. For three sample (predicted value: 227 °C, 196 °C, and 187 °C), the descriptor (NumRotatableBonds) was predominantly responsible for reducing the T_g_. When the value of NumRotatableBonds was more than 9, it could decrease T_g_. The descriptors such as NumRotatableBonds, BCUT2D_MWLOW, and PEOE_VAS13, exhibited a synergistic interaction, collectively driving the prediction below the baseline.

Analysis of the above SHAP plots for the CATB model revealed that the NumRotatableBonds descriptor exerted a more significant impact on T_g_ compared to other descriptors, which is a molecular descriptor proposed for the quantification of molecular flexibility. This descriptor belongs to the structural feature of the molecule repeat units, reflecting the number of rotatable bonds in the molecule. It is commonly associated with the presence of single bonds in the molecule, typically carbon sp^3^-carbon sp^3^. The unrestricted rotation of atoms around single bonds allows the molecule to assume various conformations, increasing the molecular flexibility as the number of single bonds rises [71]. When considering in conjunction with the SHAP dependence plot (Figure 8c), it became apparent that T_g_ and NumRotatableBonds exhibited a negative correlation. As the NumRotatableBonds value increased, molecular flexibility increased, and T_g_ consistently decreased. To further explore the chemical implication of this descriptor, we visualized its conceptual map in Figure 8d. This descriptor can be easily designed in a chemical structure. Thereby, when designing high T_g_ PI, it is advisable to keep the value of NumRotatableBonds as small as possible and increase the number of rigid bonds as well as ring bridges to improve the T_g_ value. For example, the introduction of flexible chains (e.g., -O-, -CH_2_-, -S-) and semi-flexible units (e.g., -SO_2_-) can lead to a decrease in the T_g_ of PI [72]. From the perspective of functional groups, high T_g_ usually correlates to heteroaromatic units, rigid aromatic units, or inflexible linkages [73]. We can adjust the T_g_ to the desired value by introducing the aliphatic structures or flexible linkage bonds into the PI backbone. Fr_unbrch_alkane descriptor means the number of unbranched alkanes of at least four members (excludes halogenated alkanes). The inclusion of unbranched alkane segments comprising four or more carbon atoms significantly depresses the T_g_ of polymers. This effect originates from the enhanced conformational flexibility of linear alkyl chains (e.g., -(CH_2_)*_n_*-, *n* ≥ 4), thereby facilitating segmental mobility and reducing the activation energy required for cooperative chain motion. Concomitantly, these flexible moieties expand free volume fractions while diminishing intermolecular cohesion, collectively lowering the thermal threshold for the glass-to-rubber transition. All in all, the results of the SHAP analysis showed that molecular flexibility was the key factor affecting T_g_. These interpretations supported the existing PI design theories. However, the NumRotatableBonds is just one descriptor that has a pronounced impact on T_g_. T_g_ is also influenced by several other functional groups or steric factors. The rigidity and mobility of the entire chain segment after polymerization should also be considered. Furthermore, because each descriptor does not affect the property independently, there are synergistic effects between the descriptors. The presence of synergistic effects with other descriptors may lead the same descriptor to make two opposite contributions to the property in different polymers. Therefore, the effect of each descriptor on the property is not invariant. The contribution of each descriptor should be generally approximated by combining the influential mean value over the entire dataset.

### 3.4. MD Validation

**Figure 10:**
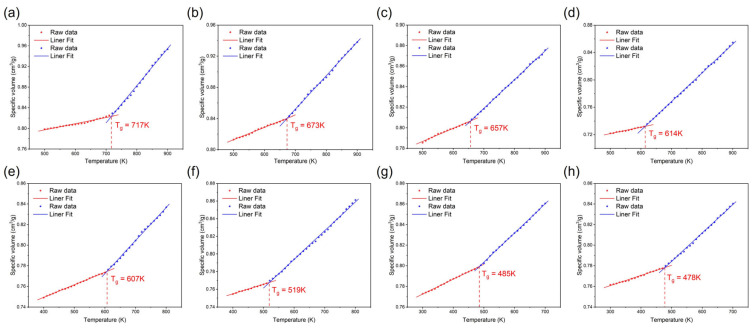
Specific volume–temperature curves of (**a**) PI-1, (**b**) PI-2, (**c**) PI-3, (**d**) PI-4, (**e**) PI-5, (**f**) PI-6, (**g**) PI-7, and (**h**) PI-8 obtained from MD simulations.

**Figure 11:**
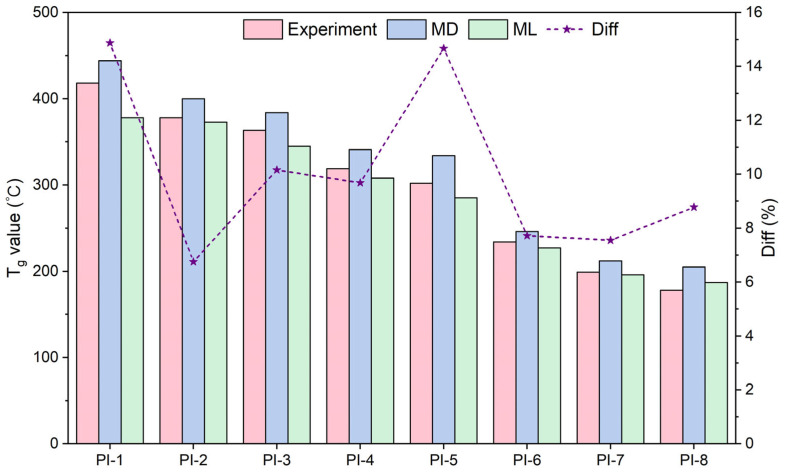
Comparison of experiment values, MD simulations, and ML predictions of eight real PIs.

We calculated descriptors for eight types of PIs using RDKit and used the CATB model to predict T_g_ values. Detailed information about their descriptors can be found in the Supplementary Materials. To validate the thermal property of the above PIs, we carried out an all-atom MD simulation to analyze their T_g_. We built the all-atoms model to simulate the eight PIs made of two components (dianhydride + diamine). To obtain the T_g_ of these systems, we simulated the cooling process by gradually decreasing the temperature from 900 K to 300 K in decrements of 10 K. The density of the system was balanced at different temperatures through 500 ps NPT MD operation under the COMPASS III force field. We obtained the average density of each system and calculated the specific volume. As the temperature decreased and approached T_g_ from above, polymer chain segments lost sufficient thermal energy to sustain large-scale cooperative motions. The significant reduction in segmental mobility led to a corresponding decrease in the rate of free volume expansion. The materials evolved from a rubbery state to a rigid glassy state. By measuring the specific volume of the PI at different temperatures, a discontinuous change interval can be found on the specific volume–temperature graph, which corresponds to the T_g_. Therefore, the scatter plots of the specific volume versus temperature were created using Origin 2024 software and subjected to two linear fits. Figure 10 plots the specific volume–temperature curves for eight PIs, displaying two distinct linear regimes corresponding to the glassy state (low temperature) and rubbery state (high temperature). Two least-square linear fit curves had constant slopes. The intersection points of two curves defined T_g_. In general, it was worth noting that the T_g_ in the simulation may be higher than the T_g_ in the experiment. This was because the time scale of MD simulation was around nanoseconds, so the modeled cooling rate was much faster than that of experiments. Although the simulated cooling rate was not exactly consistent with experiments, the T_g_ estimated from the MD simulation was still proven to reasonably agreed with the experimental value. MD simulations and experimental measurements of eight real PIs were both consistent with ML predictions. The detailed MD-calculated and ML-predicted results and experimental values collected from literature are shown in Table 3. As shown in Figure 11, the percentage error (Diff) between the calculated and predicted values in T_g_ prediction ranged from 6.75 to 14.87% within an acceptable range, considering the uncertainties in MD simulation and ML prediction. A positive value denotes that the ML prediction is lower than the MD reference (underestimation), and a negative value denotes that ML exceeds MD (overestimation). Among them, PI-2 had the best prediction with a Diff of only 6.75%. The two methods demonstrated good agreement, further validating the effectiveness and reliability of the ML models in predicting material properties. These investigations showed that the high accuracy of the developed T_g_-QSPR model had reached the level of the traditional molecular simulation, or even better, but the time consumption and hold-up computing resource were tremendously reduced.

As shown in Table 3, PI-1-PI-8 exhibited distinct NumRotatableBonds values. A higher NumRotatableBonds indicates that the molecule has more conformational freedom, which may reduce T_g_. Based on the relationship between the important descriptor and T_g_ values, PIs with higher T_g_ have been shown to have smaller NumRotatableBonds values. Among these eight PIs, PI-1 showed the largest T_g_ due to the negative contribution of NumRotatableBonds. For PI-1 and PI-2, which utilized the same diamine and different dianhydrides for polymerization, the identical NumRotatableBonds values resulted in similar predicted T_g_, while differences in experimental values stemmed from other descriptors. Flexible unit (-S-) reduced T_g_ more significantly than semi-flexible unit (-SO_2_-), which was consistent with the known theory. Commonly, more aromatic rings in the molecular chain were conducive to increasing T_g_. However, from PI-1 to PI-8, the PI molecular structure contained more benzene rings, while more ether bonds and T_g_ decreased gradually. All results consistently proved that the increasing effect of introducing a benzene ring on T_g_ did not offset the decreasing effect of a flexible bond (such as an ether bond) on T_g_. In short, the presence of rotatable bonds increased the flexibility of the molecular chain and thus decreased T_g_. The larger the proportion of rotatable bonds, the lower the T_g_. The number of rotatable bonds had a greater effect on T_g_ than the number of rings. Also, the values of T_g_ decreased significantly with the introduction of -CF_3_ for the disruption of molecular chain packing. While T_g_ cannot be solely determined based on a single descriptor and is influenced by other descriptors, the NumRotatableBonds descriptor exerts the most significant impact on T_g_, offering new insights for designing PI structures with high T_g_.

## 4. Conclusions

This study systematically explored the prediction and design of PI materials with high heat resistance, focusing on T_g_ by integrating literature data, MD simulation, and ML prediction. Our developed model not only had good predictive accuracy, but also had clear physical explanations. Based on a large volume of literature data, we applied interpretable ML techniques to identify key descriptors that significantly influenced the T_g_ of PI copolymers. These findings provided the crucial theoretical guidance for the design of novel heat-resistant materials. PI structures collected from the literature were first processed using three main steps after data acquisition: converting from molecular structures to mechanically configurable SMILES strings, extracting their molecular descriptors, and simplifying the number of descriptions by comparing three methods to ultimately form 43 key descriptors. After systematically comparing nine popular ML algorithms, all models showed high predictability and stability. The T_g_-QSPR model trained by CATB algorithm had the highest accuracy, with an R^2^ score of 0.895, MAE of 18.58 °C, and RMSE of 23.06 °C on the test set. Based on the SHAP analysis, the physicochemical meaning of the key descriptors was carefully analyzed and interpreted. The descriptor corresponding to chain flexibility (NumRotatableBonds) had a significant negative impact on T_g_. Finally, we used MD simulation to calculate eight types of PI structures with distinct T_g_ values that were not present in the dataset to validate the accuracy of the trained model. A comparison of the ML-predicted results with the MD-calculated results revealed some errors, with Diff as low as 6.75%, indicating the high precision of the model. Within its applicability domain, this ML technology provided an impressive acceleration over the MD simulation. The T_g_ of thousands of polymers can be obtained within seconds, whereas it would have taken a longer time, even years, to simulate them. The utilization of large-scale and diverse datasets for predictive modeling has been proven to yield more accurate and stable models, as well as improve model interpretability. Importantly, such approach is not limited to PI. It can be applied to many categories of materials. It further emphasizes the enormous potential of mixed strategies combining data-driven and MD simulation for targeted applications, as implemented in this work. In addition, the work undertaken here could be further refined and improved by future efforts in generating additional data or improving data quality. Collecting molecular structures containing macrocyclic or other complex structures and their corresponding properties can further expand the chemical space learned by the model to improve the accuracy of the model in predicting the properties of PI. Exploring more complex and advanced molecular representations and combining other types of feature representations (such as Morgan fingerprints and graphs) can supplement richer structural information to improve model interpretability. It should be noted that the T_g_ of PI is currently predicted only by their chemical structures in this work, though parameters such as measurement conditions, molecular weight, and their polydispersity and degree of crystallinity after polymerization also have an impact on T_g_. In the future studies, we will collect relevant data and optimize ML models. For some unreasonable structures in the design results, we will continue to optimize the molecular structures, use Schuffenhauer’s synthetic accessibility scores to calculate their synthesizability, and hope to receive further suggestions and revisions. The combination of structure screening and exploring the process parameters of synthesizing these potential polymers provides the possibility for the final synthesis of high-performance polymers.

## Figures and Tables

**Table 1:** Performance statistics for T_g_ prediction by different feature selection methods (taking XGB as an example).

Selection Method	Training Set			Test Set			10-Fold Cross-Validation ^a^		
	R^2^	MAE (°C)	RMSE (°C)	R^2^	MAE (°C)	RMSE (°C)	R^2^	MAE (°C)	RMSE (°C)
Feature importance	0.966	9.48	12.65	0.801	22.69	31.69	0.808 ± 0.022	21.48 ± 1.83	30.81 ± 2.42
Mutual_info_regression	0.974	8.25	11.05	0.797	23.32	31.99	0.785 ± 0.031	22.29 ± 2.25	32.68 ± 2.83
LASSO regularization	0.968	9.21	12.39	0.789	24.21	32.62	0.778 ± 0.036	25.10 ± 2.51	33.56 ± 3.36

^a^ The error is the standard deviation of the cross-validation results.

**Table 2:** Comparison of T_g_-QSPR models using different algorithms.

Models	Training Set			Test Set			10-Fold Cross-Validation		
	R^2^	MAE (°C)	RMSE (°C)	R^2^	MAE (°C)	RMSE (°C)	R^2^	MAE (°C)	RMSE (°C)
XGB	0.989	5.27	7.10	0.814	21.80	30.67	0.802 ± 0.029	22.30 ± 1.92	31.28 ± 2.81
ANN	0.949	11.28	15.59	0.783	23.88	33.09	0.774 ± 0.041	24.15 ± 2.34	32.26 ± 3.24
DNN	0.978	7.23	10.12	0.849	20.09	27.60	0.834 ± 0.027	19.82 ± 1.83	28.15 ± 2.54
ET	0.985	5.36	8.37	0.800	23.26	31.94	0.790 ± 0.028	22.86 ± 1.96	32.11 ± 2.78
GBDT	0.991	5.03	6.53	0.792	22.91	32.44	0.784 ± 0.036	23.78 ± 2.12	33.12 ± 3.16
LGBM	0.988	5.39	7.55	0.808	22.15	31.16	0.813 ± 0.031	23.35 ± 2.06	30.78 ± 3.11
AB	0.988	5.13	7.56	0.800	23.28	31.86	0.796 ± 0.029	22.56 ± 1.61	29.65 ± 3.02
CATB	0.989	5.37	7.08	0.895	18.58	23.06	0.901 ± 0.025	17.91 ± 1.31	23.21 ± 2.29
GPR	0.950	10.83	15.49	0.802	22.42	31.60	0.809 ± 0.026	23.49 ± 1.78	32.34 ± 2.44

**Table 3:** Experimental characterization, MD simulation, and model prediction values of PI films.

Name	NumRotatableBonds	ML (°C)	MD (°C)	Experiment (°C)	Diff (%)	Ref.
PI-1	3	378	444	418	14.87	[61]
PI-2	3	373	400	378	6.75	[61]
PI-3	4	345	384	363.5	10.16	[62]
PI-4	5	308	341	319	9.68	[63]
PI-5	8	285	334	302	14.67	[64]
PI-6	9	227	246	234	7.72	[65]
PI-7	9	196	212	199	7.55	[66]
PI-8	11	187	205	178	8.78	[67]

Note: Diff = [(MD − ML)/MD] × 100%.

## Data Availability

Data is contained within the article or Supplementary Materials.

## Supplementary Materials

The following supporting information can be downloaded at: https://www.mdpi.com/article/10.3390/polym17152083/s1. Table S1. Reference links to literature on polyimide structures and property data; Table S2. Explanation of 210 descriptors, which were calculated by RDKit Python software package; Figure S1. The prediction performance of ML models using the feature importance method; Figure S2. The prediction performance of ML models using the mutual_info_regression method; Figure S3. The prediction performance of ML models using the LASSO regularization method; Note S1. Detailed optimal parameter settings of different models; Excel sheet1 PI structures and SMILES; Excel sheet2 SMILES normalization; Excel sheet3 All molecular descriptors; Excel sheet 4 Removing zero column; Excel sheet 5–7 Selected descriptors using three feature filtering methods; Excel sheet 8 Molecular descriptors for PI-1 to PI-8.

## Abbreviations

The following abbreviations are used in this manuscript:

PI	polyimide
T_g_	glass transition temperature
ML	machine learning
MD	molecular dynamics
QSPR	quantitative structure–property relationships
LASSO	Least Absolute Shrinkage and Selection Operator
XGB	eXtreme Gradient Boosting
ANN	Artificial Neural Network
DNN	Deep Neural Network
ET	Extra Trees
GBDT	Gradient Boosting Decision Tree
LGBM	Light Gradient Boosting Machine
AB	AdaBoost regression
CATB	Categorical Boosting
GPR	Gaussian Process Regression
R^2^	coefficient of determination
MAE	mean absolute error
RMSE	root mean square error
SHAP	SHapley Additive exPlanations
