# Supplementary material for: Prediction and Interpretability Study of the Glass Transition Temperature of Polyimide Based on Machine Learning and Molecular Dynamics Simulations
Source: Polymers (Basel). 2025 Jul 30;17(15):2083. doi: 10.3390/polym17152083 (PMC12349611; doi:10.3390/polym17152083)
Supplement: Supplementary file 1 [file polymers-17-02083-s001.zip › polymers-3772057-Supplementary Materials.pdf]

polymers

MDPI

Supplementary Materials

Table S1. Reference links to literature on polyimide structures and property data.

Reference ID	DOI
1	10.1002/app.34914
2	10.1016/0032-3861(70)90066-2
3	10.1002/app.1985.070300611
4	10.1002/pola.1991.080290816
5	10.1002/pi.4980020507
6	10.1002/pol.1972.150100619
7	10.1002/pola.1988.080260813
8	10.1039/jm9960601459
9	10.1016/1381-5148(96)00020-X
10	10.1002/apmc.1986.051450116
11	10.1002/polb.1991.090290308
12	10.1002/(SICI)1099-0518(199808)36:11<1791::AID-POLA13>3.0.CO;2-9
13	10.1021/ma00093a011
14	10.1021/ma00181a002
15	10.1002/(SICI)1099-0518(199706)35:8<1527::AID-POLA21>3.0.CO;2-8
16	10.1002/(SICI)1099-0518(19960415)34:5<747::AID-POLA4>3.0.CO;2-K
17	10.1002/pola.1994.080321020
18	10.1002/pola.1994.080321218
19	10.1002/app.1995.070581102
20	10.1016/0014-3057(95)00196-4
21	10.1088/0954-0083/9/3/002
22	10.1021/cm010088v
23	10.1002/(SICI)1097-4628(19970214)63:7<865::AID-APP6>3.0.CO;2-O
24	10.1002/pol.1977.170150629
25	10.1002/polc.5070740111
26	10.1016/S0032-3861(00)00610-8
27	10.1002/polb.1992.090300813
28	10.1002/app.25289
29	10.1002/pola.1993.080311224
30	10.1002/pola.1993.080311117
31	10.1021/ma00070a022
32	10.1002/app.28715
33	10.1080/15685551.2013.840514
34	10.1080/10601325.2014.906261
35	10.1002/(SICI)1099-0518(19990315)37:6<805::AID-POLA16>3.0.CO;2-9
36	10.1007/s10965-012-0053-9
37	10.1016/j.reactfunctpolym.2011.05.011
38	10.1007/BF03246107
39	10.1007/s00289-012-0858-z
40	10.1016/j.polymdegradstab.2011.03.018
41	10.1002/app.28118
42	10.1016/j.polymdegradstab.2008.01.005
43	10.1002/pat.1072
44	10.1002/pat.3062
45	10.1002/pi.3184
46	10.1002/app.36650
47	10.1007/s00289-010-0441-4
48	10.1088/0954-0083/12/1/313
49	10.1002/pol.1978.170160120
50	10.1021/ma9512566

51	10.1002/app.31523
52	10.1021/ma8022475
53	10.1021/ma802305q
54	10.1080/10587250008025674
55	10.1002/app.32213
56	10.1002/polb.1995.090330423
57	10.1002/pola.21411
58	10.1016/0032-3861(95)90669-S
59	10.1016/j.eurpolymj.2010.01.020
60	10.1002/pat.1519
61	10.1002/app.29868
62	10.1016/S0032-3861(05)80027-8
63	10.1016/S0032-3861(97)00114-6
64	10.1039/C4NJ02105C
65	10.1021/ma0524732
66	10.1002/polb.23779
67	10.1002/app.20287
68	10.1002/app.23580
69	10.1002/pola.1992.080300723
70	10.1021/ma902013y
71	10.1163/1568555054460033
72	10.1081/PPT-120029979
73	10.1021/acs.macromol.5b00183
74	10.1002/app.32668
75	10.1177/0954008312436704
76	10.1080/15685551.2013.840473
77	10.1080/10601325.2013.780948
78	10.1002/pola.23183
79	10.1038/pj.2011.28
80	10.1016/S0032-3861(01)00678-4
81	10.1039/c3ta10261k
82	10.1088/0954-0083/8/3/009
83	10.1002/(SICI)1522-9505(19980201)254:1<67::AID-APMC67>3.0.CO;2-1
84	10.1081/MA-100101162
85	10.1177/0954008308090458
86	10.1557/PROC-227-3
87	10.1021/ma302456h
88	10.1002/macp.1993.021941019
89	10.1002/app.23627
90	10.1177/0954008307077769
91	10.1021/ma961079o
92	10.1016/S0014-3057(01)00229-4
93	10.1002/pola.1991.080290318
94	10.1002/macp.1990.021910113
95	10.1007/s003960050531
96	10.1002/pola.10105
97	10.1007/s10118-011-1058-2
98	10.1002/app.11885
99	10.1021/ma00183a009
100	10.1002/macp.1997.021980224
101	10.1021/ma00093a011
102	10.1016/j.reactfunctpolym.2014.09.018
103	10.1163/15685610152715782
104	10.1080/10587259908025991
105	10.1016/j.polymdegradstab.2010.05.002
106	10.1016/j.polymdegradstab.2010.07.025
107	10.1002/pi.2979
108	10.1016/j.polymer.2007.12.010
109	10.1021/bk-1996-0632.ch009
110	10.1021/ma0214557

111	10.1016/S0032-3861(99)00723-5
112	10.1163/156855511X615678
113	10.1002/pola.21087
114	10.1080/10601329808000557
115	10.1002/macp.1993.021940415
116	10.1016/j.polymer.2015.12.008
117	10.1021/ma0017485
118	10.1016/j.eurpolymj.2008.08.013
119	10.1002/pola.1993.080310409
120	10.1002/pola.10992
121	10.1007/s002890050270
122	10.1002/pola.20165
123	10.1021/ma034445u
124	10.1021/la0348158
125	10.1016/S0032-3861(02)00530-X
126	1016/j.polymer.2005.12.074
127	10.1016/j.polymer.2013.08.014
128	10.1177/0954008305044460
129	10.1002/pola.10918
130	10.1002/(SICI)1097-4628(19960801)61:5<741::AID-APP4>3.0.CO;2-O
131	10.1002/pola.21241
132	10.1002/pola.10338
133	10.1002/app.20173
134	10.1016/S0032-3861(01)00287-7
135	10.1002/pola.10264
136	10.1002/(SICI)1521-3935(19991001)200:10<2402::AID-MACP2402>3.0.CO;2-C
137	10.1016/j.jssc.2004.12.010
138	10.1021/cm071430s
139	10.1002/pola.22308
140	10.1177/0954008306056751
141	10.1177/0954008306063639
142	10.1016/j.polymer.2015.11.051
143	10.1016/j.synthmet.2013.08.009
144	10.1007/s00396-010-2202-1
145	10.1007/s12034-013-0542-4
146	10.1016/0032-3861(94)90747-1
147	10.1002/app.24800
148	10.1002/(SICI)1099-0488(19990715)37:14<1633::AID-POLB8>3.0.CO;2-#
149	10.1002/pol.1984.170220507
150	10.1002/(SICI)1099-0518(19990915)37:18<3646::AID-POLA13>3.0.CO;2-H
151	10.1002/pen.22059
152	10.1038/pj.2011.67
153	10.1295/polymj.32.948
154	10.1016/0032-3861(93)90368-K
155	10.1002/pola.1994.080321012
156	10.1002/app.21620
157	10.1163/156856103322396730
158	10.1002/app.30601
159	10.1002/(SICI)1099-0518(19970430)35:6<1149::AID-POLA18>3.0.CO;2-6
160	10.1002/app.21859
161	10.1002/pola.10010
162	10.1021/ma00091a003
163	10.1080/10587259808044459
164	10.1021/ma00112a003
165	10.1002/polb.1995.090330317
166	10.1002/pen.24268
167	10.3144/expresspolymlett.2008.95
168	10.1002/pen.10875
169	10.1016/S0014-3057(98)00001-9
170	10.1002/pat.3844

171	10.1021/ma970530o
172	10.1002/app.29388
173	10.1088/0954-0083/7/3/011
174	10.1002/macp.1992.021930716
175	10.1002/macp.1996.021970617
176	10.1002/app.36956
177	10.1016/S0014-3057(00)00229-9
178	10.1002/marc.1997.030180402
179	10.1295/polymj.34.158
180	10.1295/polymj.34.601
181	10.1088/0954-0083/10/1/017
182	10.1080/00222348.2012.661665
183	10.1080/10601329708014998
184	10.2478/s11532-006-0029-x
185	10.1016/j.synthmet.2012.01.016
186	10.1117/12.585015
187	10.1016/j.polymer.2004.07.053
188	10.1002/app.33202
189	10.1021/jp904250r
190	10.1117/12.585018
191	10.1364/JOSAB.13.001713
192	10.1002/pola.21777
193	10.1002/pola.21833
194	10.1002/(SICI)1097-4628(19991205)74:10<2404::AID-APP8>3.0.CO;2-Z
195	10.1002/1439-2054(20010701)286:7<434::AID-MAME434>3.0.CO;2-F
196	10.1295/polymj.PJ2008076
197	10.1002/polb.1993.090310804
198	10.1002/polb.1989.090270908
199	10.1007/s00396-014-3248-2
200	10.1002/app.32007
201	10.1016/j.polymer.2006.04.001
202	10.1002/polb.1993.090310908
203	10.1002/app.31761
204	10.1007/s00289-015-1537-7
205	10.1021/ma010615w
206	10.1080/15685551.2013.840497
207	10.1177/0954008310381929
208	10.1002/pat.1633
209	10.1295/koron.68.24
210	10.1016/S0032-3861(00)00761-8
211	10.1016/S0032-3861(99)00420-6
212	10.1016/j.matchemphys.2013.10.012
213	10.1016/S0032-3861(99)00613-8
214	10.1080/10236660211185
215	10.1002/app.20677
216	10.1002/app.22673
217	10.1038/s41467-023-38145-w
218	10.1021/ma001821r
219	10.1021/ma971374o
220	10.1080/10587259808044455
221	10.1002/app.25504
222	10.1080/00222348.2011.557621
223	10.1002/macp.1993.021941111
224	10.1002/pola.1992.080300908
225	10.1295/polymj.35.662
226	10.1007/s003960100573
227	10.1023/A:1026034213156
228	10.1002/app.11265
229	10.1016/j.polymdegradstab.2010.04.003
230	10.1007/s10965-014-0572-7

231	10.1080/10601329808000993
232	10.1002/app.28524
233	10.1007/s13233-012-0007-4
234	10.1007/s10971-023-06143-5
235	10.1177/0954008307078514
236	10.1016/j.polymer.2005.10.003
237	10.1016/j.polymer.2007.07.001
238	10.1016/j.polymdegradstab.2010.03.026

Table S2. Explanation of 210 descriptors which were calculated by RDKit Python software package.

ID	Descriptor Name	Description
1	MaxAbsEStateIndex	Maximum absolute EState index
2	MaxEStateIndex	Maximum EState index
3	MinAbsEStateIndex	Minimum absolute EState index
4	MinEStateIndex	Minimum EState index
5	qed	Calculate the weighted sum of ADS mapped properties
6	SPS	Shortest Path Sum
7	MolWt	The average molecular weight of the molecule
8	HeavyAtomMolWt	The average molecular weight of the molecule ignoring hydrogens
9	ExactMolWt	The exact molecular weight of the molecule
10	NumValenceElectrons	The number of valence electrons the molecule has
11	NumRadicalElectrons	The number of radical electrons the molecule has (says nothing about spin state)
12	MaxPartialCharge	Maximum Partial Charge. A partial charge is a non-integer charge value when measured in elementary charge units
13	MinPartialCharge	Minimum Partial Charge. A partial charge is a non-integer charge value when measured in elementary charge units
14	MaxAbsPartialCharge	Maximum absolute Partial Charge. A partial charge is a non-integer charge value when measured in elementary charge units
15	MinAbsPartialCharge	Minimum absolute Partial Charge. A partial charge is a non-integer charge value when measured in elementary charge units
16	FpDensityMorgan1	Morgan fingerprint, radius 1
17	FpDensityMorgan2	Morgan fingerprint, radius 2
18	FpDensityMorgan3	Morgan fingerprint, radius 3
19	BCUT2D_MWHI	Implements BCUT descriptors From J. Chem. Inf. Comput. Sci., Vol. 39, No. 1, 1999
20	BCUT2D_MWLOW	Implements BCUT descriptors From J. Chem. Inf. Comput. Sci., Vol. 39, No. 1, 1999
21	BCUT2D_CHGHI	Implements BCUT descriptors From J. Chem. Inf. Comput. Sci., Vol. 39, No. 1, 1999
22	BCUT2D_CHGLO	Implements BCUT descriptors From J. Chem. Inf. Comput. Sci., Vol. 39, No. 1, 1999
23	BCUT2D_LOGPHI	Implements BCUT descriptors From J. Chem. Inf. Comput. Sci., Vol. 39, No. 1, 1999
24	BCUT2D_LOGPLOW	Implements BCUT descriptors From J. Chem. Inf. Comput. Sci., Vol. 39, No. 1, 1999
25	BCUT2D_MRHI	Implements BCUT descriptors From J. Chem. Inf. Comput. Sci., Vol. 39, No. 1, 1999
26	BCUT2D_MRLOW	Implements BCUT descriptors From J. Chem. Inf. Comput. Sci., Vol. 39, No. 1, 1999
27	AvgIpc	Average information content of the path lengths
28	BalabanJ	Balaban J index characterizing molecular topology
29	BertzCT	A topological index meant to quantify “complexity” of molecules.

30	Chi0	From equations (1),(9) and (10) of Rev. Comp. Chem. vol 2, 367-422, (1991)
31	Chi0n	Similar to Hall Kier Chi0v, but uses nVal instead of valence. This makes a big difference after we get out of the first row
32	Chi0v	From equations (5),(9) and (10) of Rev. Comp. Chem. vol 2, 367-422, (1991)
33	Chi1	From equations (1),(11) and (12) of Rev. Comp. Chem. vol 2, 367-422, (1991)
34	Chi1n	Similar to Hall Kier Chi1v, but uses nVal instead of valence
35	Chi1v	From equations (5),(11) and (12) of Rev. Comp. Chem. vol 2, 367-422, (1991)
36	Chi2n	Similar to Hall Kier Chi2v, but uses nVal instead of valence. This makes a big difference after we get out of the first row
37	Chi2v	From equations (5), (15) and (16) of Rev. Comp. Chem. vol 2, 367-422, (1991)
38	Chi3n	Similar to Hall Kier Chi3v, but uses nVal instead of valence. This makes a big difference after we get out of the first row
39	Chi3v	From equations (5),(15) and (16) of Rev. Comp. Chem. vol 2, 367-422, (1991)
40	Chi4n	Similar to Hall Kier Chi4v, but uses nVal instead of valence. This makes a big difference after we get out of the first row
41	Chi4v	From equations (5),(15) and (16) of Rev. Comp. Chem. vol 2, 367-422, (1991)
42	HallKierAlpha	The Hall-Kier alpha value for a molecule
43	Ipc	The information content of the coefficients of the char-acteristic polynomial of the adjacency matrix of a hy-drogensuppressed graph of a molecule
44	Kappa1	Hall-Kier Kappa1 value
45	Kappa2	Hall-Kier Kappa2 value
46	Kappa3	Hall-Kier Kappa3 val
47	LabuteASA	Labute's Approximate Surface Area
48	PEOE_VSA1	MOE Charge VSA Descriptor 1 (-inf < x < -0.30)
49	PEOE_VSA10	MOE Charge VSA Descriptor 10 (0.10 <= x < 0.15)
50	PEOE_VSA11	MOE Charge VSA Descriptor 11 (0.15 <= x < 0.20)
51	PEOE_VSA12	MOE Charge VSA Descriptor 12 (0.20 <= x < 0.25)
52	PEOE_VSA13	MOE Charge VSA Descriptor 13 (0.25 <= x < 0.30)
53	PEOE_VSA14	MOE Charge VSA Descriptor 14 (0.30 <= x < inf)
54	PEOE_VSA2	MOE Charge VSA Descriptor 2 (-0.30 <= x < -0.25)
55	PEOE_VSA3	MOE Charge VSA Descriptor 3 (-0.25 <= x < -0.20)
56	PEOE_VSA4	MOE Charge VSA Descriptor 4 (-0.20 <= x < -0.15)
57	PEOE_VSA5	MOE Charge VSA Descriptor 5 (-0.15 <= x < -0.10)
58	PEOE_VSA6	MOE Charge VSA Descriptor 6 (-0.10 <= x < -0.05)
59	PEOE_VSA7	MOE Charge VSA Descriptor 7 (-0.05 <= x < 0.00)
60	PEOE_VSA8	MOE Charge VSA Descriptor 8 (0.00 <= x < 0.05)
61	PEOE_VSA9	MOE Charge VSA Descriptor 9 (0.05 <= x < 0.10)
62	SMR_VSA1	MOE MR VSA Descriptor 1 (-inf < x < 1.29)
63	SMR_VSA10	MOE MR VSA Descriptor 10 (4.00 <= x < inf)
64	SMR_VSA2	MOE MR VSA Descriptor 2 (1.29 <= x < 1.82)
65	SMR_VSA3	MOE MR VSA Descriptor 3 (1.82 <= x < 2.24)
66	SMR_VSA4	MOE MR VSA Descriptor 4 (2.24 <= x < 2.45)
67	SMR_VSA5	MOE MR VSA Descriptor 5 (2.45 <= x < 2.75)
68	SMR_VSA6	MOE MR VSA Descriptor 6 (2.75 <= x < 3.05)
69	SMR_VSA7	MOE MR VSA Descriptor 7 (3.05 <= x < 3.63)
70	SMR_VSA8	MOE MR VSA Descriptor 8 (3.63 <= x < 3.80)
71	SMR_VSA9	MOE MR VSA Descriptor 9 (3.80 <= x < 4.00)
72	SlogP_VSA1	MOE logP VSA Descriptor 1 (-inf < x < -0.40)

73	SlogP_VSA10	MOE logP VSA Descriptor 10 (0.40 <= x < 0.50)
74	SlogP_VSA11	MOE logP VSA Descriptor 11 (0.50 <= x < 0.60)
75	SlogP_VSA12	MOE logP VSA Descriptor 12 (0.60 <= x < inf)
76	SlogP_VSA2	MOE logP VSA Descriptor 2 (-0.40 <= x < -0.20)
77	SlogP_VSA3	MOE logP VSA Descriptor 3 (-0.20 <= x < 0.00)
78	SlogP_VSA4	MOE logP VSA Descriptor 4 (0.00 <= x < 0.10)
79	SlogP_VSA5	MOE logP VSA Descriptor 5 (0.10 <= x < 0.15)
80	SlogP_VSA6	MOE logP VSA Descriptor 6 (0.15 <= x < 0.20)
81	SlogP_VSA7	MOE logP VSA Descriptor 7 (0.20 <= x < 0.25)
82	SlogP_VSA8	MOE logP VSA Descriptor 8 (0.25 <= x < 0.30)
83	SlogP_VSA9	MOE logP VSA Descriptor 9 (0.30 <= x < 0.40)
84	TPSA	The polar surface area of a molecule based upon fragments
85	EState_VSA1	EState VSA Descriptor 1 (-inf < x < -0.39)
86	EState_VSA10	EState VSA Descriptor 10 (9.17 <= x < 15.00)
87	EState_VSA11	EState VSA Descriptor 11 (15.00 <= x < inf)
88	EState_VSA2	EState VSA Descriptor 2 (-0.39 <= x < 0.29)
89	EState_VSA3	EState VSA Descriptor 3 (0.29 <= x < 0.72)
90	EState_VSA4	EState VSA Descriptor 4 (0.72 <= x < 1.17)
91	EState_VSA5	EState VSA Descriptor 5 (1.17 <= x < 1.54)
92	EState_VSA6	EState VSA Descriptor 6 (1.54 <= x < 1.81)
93	EState_VSA7	EState VSA Descriptor 7 (1.81 <= x < 2.05)
94	EState_VSA8	EState VSA Descriptor 8 (2.05 <= x < 4.69)
95	EState_VSA9	EState VSA Descriptor 9 (4.69 <= x < 9.17)
96	VSA_EState1	VSA EState Descriptor 1 (-inf < x < 4.78)
97	VSA_EState10	VSA EState Descriptor 10 (11.00 <= x < inf)
98	VSA_EState2	VSA EState Descriptor 2 (4.78 <= x < 5.00)
99	VSA_EState3	VSA EState Descriptor 3 (5.00 <= x < 5.41)
100	VSA_EState4	VSA EState Descriptor 4 (5.41 <= x < 5.74)
101	VSA_EState5	VSA EState Descriptor 5 (5.74 <= x < 6.00)
102	VSA_EState6	VSA EState Descriptor 6 (6.00 <= x < 6.07)
103	VSA_EState7	VSA EState Descriptor 7 (6.07 <= x < 6.45)
104	VSA_EState8	VSA EState Descriptor 8 (6.45 <= x < 7.00)
105	VSA_EState9	VSA EState Descriptor 9 (7.00 <= x < 11.00)
106	FractionCSP3	CalcFractionCSP3((Mol)mol) -> float : returns the fraction of C atoms that are SP3 hybridized
107	HeavyAtomCount	Number of heavy atoms a molecule
108	NHOHCount	Number of NHs or OHs
109	NOCount	Number of Nitrogens and Oxygens
110	NumAliphaticCarbocycles	Number of aliphatic (containing at least one non-aromatic bond) carbocycles for a molecule
111	NumAliphaticHeterocycles	Number of aliphatic (containing at least one non-aromatic bond) heterocycles for a molecule
112	NumAliphaticRings	Number of aliphatic (containing at least one non-aromatic bond) rings for a molecule
113	NumAromaticCarbocycles	Number of aromatic carbocycles for a molecule
114	NumAromaticHeterocycles	Number of aromatic heterocycles for a molecule
115	NumAromaticRings	Refers to the number of aromatic rings in a molecule
116	NumHAcceptors	Number of Hydrogen Bond Acceptors
117	NumHDonors	Number of Hydrogen Bond Donors
118	NumHeteroatoms	Number of Heteroatoms
119	NumRotatableBonds	Number of Rotatable Bonds
120	NumSaturatedCarbocycles	Number of saturated carbon rings in a molecule
121	NumSaturatedHeterocycles	Number of saturated heterocycles in a molecule
122	NumSaturatedRings	Number of saturated rings in a molecule
123	RingCount	Ring count
124	MolLogP	Wildman-Crippen LogP value
125	MolMR	Wildman-Crippen MR value
126	fr_Al_COO	Number of aliphatic carboxylic acids
127	fr_Al_OH	Number of aliphatic hydroxyl groups

128	fr_Al_OH_noTert	Number of aliphatic hydroxyl groups excluding tert-OH
129	fr_ArN	Number of N functional groups attached to aromatics
130	fr_Ar_COO	Number of Aromatic carboxylic acide
131	fr_Ar_N	Number of aromatic nitrogens
132	fr_Ar_NH	Number of aromatic amines
133	fr_Ar_OH	Number of aromatic hydroxyl groups
134	fr_COO	Number of carboxylic acids
135	fr_COO2	Number of carboxylic acids
136	fr_C_O	Number of carbonyl O
137	fr_C_O_noCOO	Number of carbonyl O, excluding COOH
138	fr_C_S	Number of thiocarbonyl
139	fr_HOCCN	Number of C(OH)CCN-Ctert-alkyl or C(OH)CCNcy-clic
140	fr_Imine	Number of Imines
141	fr_NH0	Number of Tertiary amines
142	fr_NH1	Number of Secondary amines
143	fr_NH2	Number of Primary amines
144	fr_N_O	Number of hydroxylamine groups
145	fr_Ndealkylation1	Number of XCCNR groups
146	fr_Ndealkylation2	Number of specific types of N-dealkylation sites
147	fr_Nhpyrrole	Number of H-pyrrole nitrogens
148	fr_SH	Number of thiol groups
149	fr_aldehyde	Number of aldehydes
150	fr_alkyl_carbamate	Number of alkyl carbamates (subject to hydrolysis)
151	fr_alkyl_halide	Number of alkyl halides
152	fr_allylic_oxid	Number of allylic oxidation sites excluding steroid dienone
153	fr_amide	Number of amide
154	fr_amidine	Number of amidine groups
155	fr_aniline	Number of anilines
156	fr_aryl_methyl	Number of aryl methyl sites for hydroxylation
157	fr_azide	Number of azide groups
158	fr_azo	Number of azo groups
159	fr_barbitur	Number of barbiturate groups
160	fr_benzene	Number of benzene rings
161	fr_benzodiazepine	Number of benzodiazepines with no additional fused rings
162	fr_bicyclic	Number of bicycles (bicyclic systems)
163	fr_diazo	Number of diazo groups
164	fr_dihydropyridine	Number of dihydropyridines
165	fr_epoxide	Number of epoxide rings
166	fr_ester	Number of esters
167	fr_ether	Number of ether oxygens (including phenoxy)
168	fr_furan	Number of furan rings
169	fr_guanido	Number of guanidine groups
170	fr_halogen	Number of halogen atoms
171	fr_hdrzine	Number of hydrazine groups
172	fr_hdrzone	Number of hydrazone groups
173	fr_imidazole	Number of imidazole rings
174	fr_imide	Number of imide groups
175	fr_isocyan	Number of isocyanates
176	fr_isothiocyan	Number of isothiocyanates
177	fr_ketone	Number of ketones
178	fr_ketone_Topliss	Number of ketones excluding diaryl, a,bunsat. dienones, heteroatom on Calpha
179	fr_lactam	Number of beta lactams
180	fr_lactone	Number of cyclic esters (lactones)
181	fr_methoxy	Number of methoxy groups -OCH3
182	fr_morpholine	Number of morpholine rings

183	fr_nitrile	Number of nitriles
184	fr_nitro	Number of nitro groups
185	fr_nitro_arom	Number of nitro benzene ring substituents
186	fr_nitro_arom_nonortho	Number of non-ortho nitro benzene ring substituents
187	fr_nitroso	Number of nitroso groups, excluding NO2
188	fr_oxazole	Number of oxazole rings
189	fr_oxime	Number of oxime groups
190	fr_para_hydroxylation	Number of para-hydroxylation sites
191	fr_phenol	Number of phenol
192	fr_phenol_noOrthoHbond	Number of phenolic OH excluding ortho intramolecular Hbond substituents
193	fr_phos_acid	Number of phosphoric acid groups
194	fr_phos_ester	Number of phosphoric ester groups
195	fr_piperdine	Number of piperdine rings
196	fr_piperzine	Number of piperzine rings
197	fr_priamide	Number of primary amides
198	fr_prisulfonamd	Number of primary sulfonamides
199	fr_pyridine	Number of pyridine rings
200	fr_quatN	Number of quarternary nitrogens
201	fr_sulfide	Number of thioether
202	fr_sulfonamd	Number of sulfonamides
203	fr_sulfone	Number of sulfone groups
204	fr_term_acetylene	Number of terminal acetylenes
205	fr_tetrazole	Number of tetrazole rings
206	fr_thiazole	Number of thiazole rings
207	fr_thiocyan	Number of thiocyanates
208	fr_thiophene	Number of thiophene rings
209	fr_unbrch_alkane	Number of unbranched alkanes of at least 4 members (excludes halogenated alkanes)
210	fr_urea	Number of urea groups

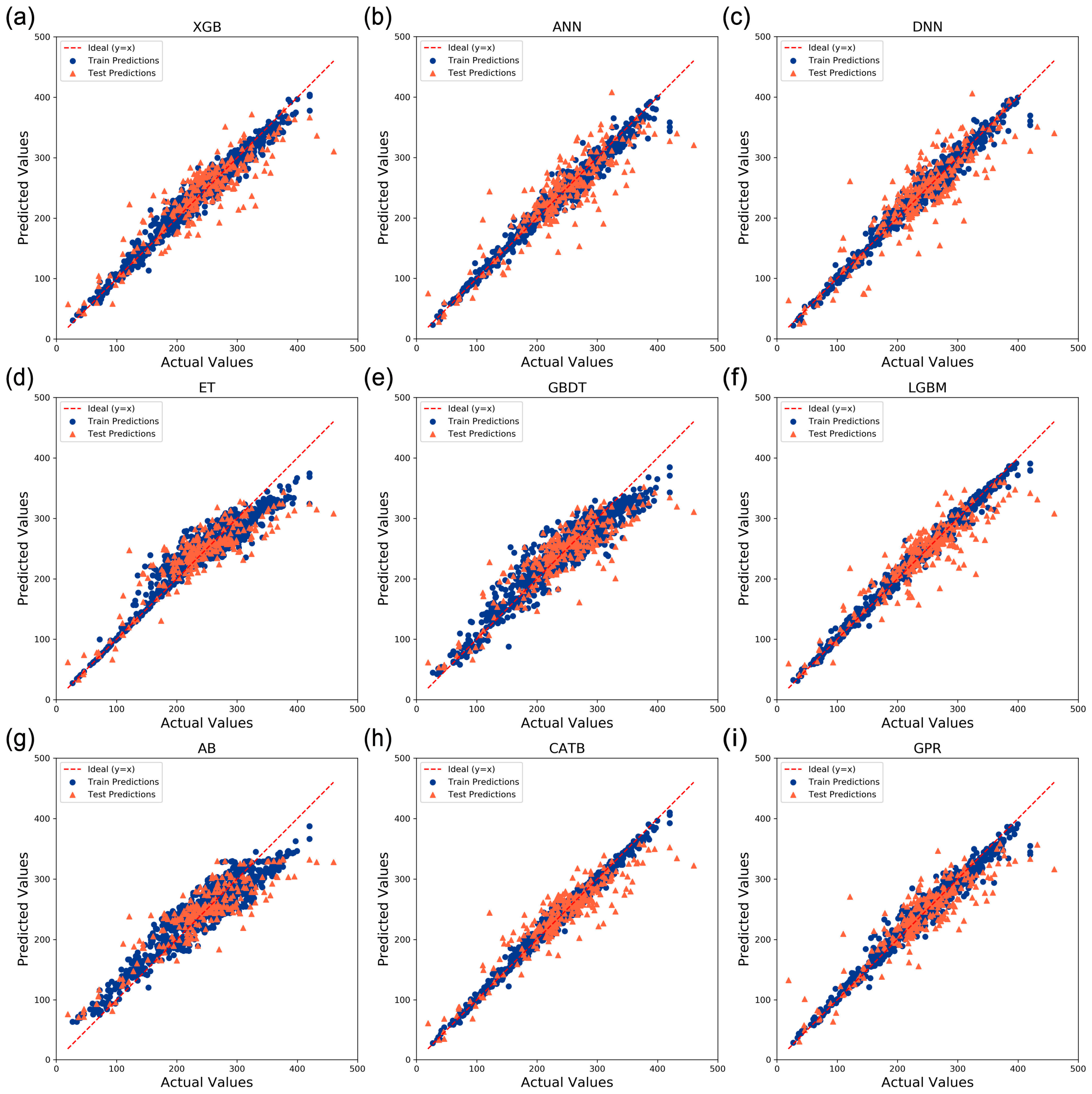

Figure S1. The prediction performance of ML models using feature importance method.

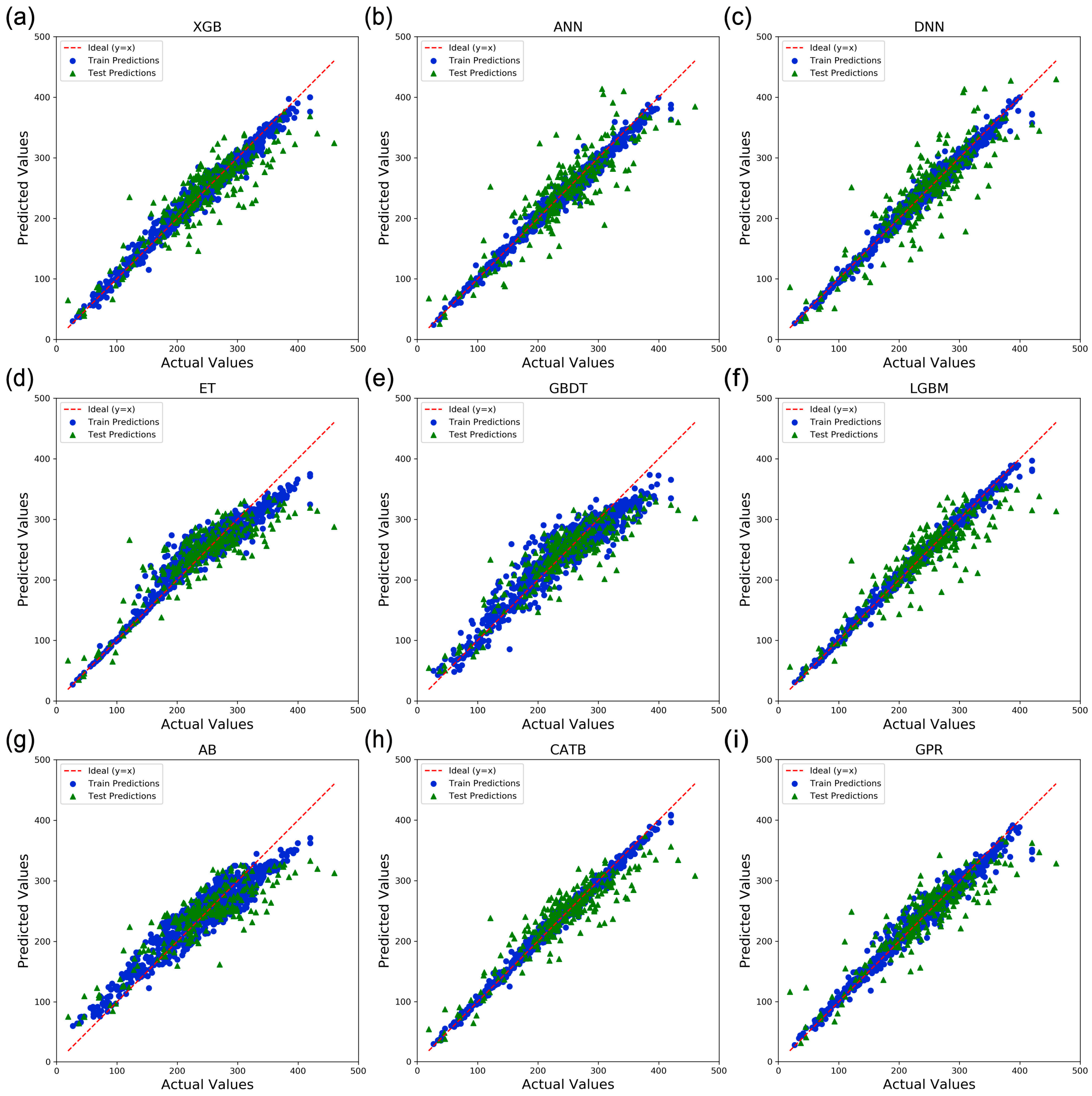

Figure S2. The prediction performance of ML models using mutual_info_regression method.

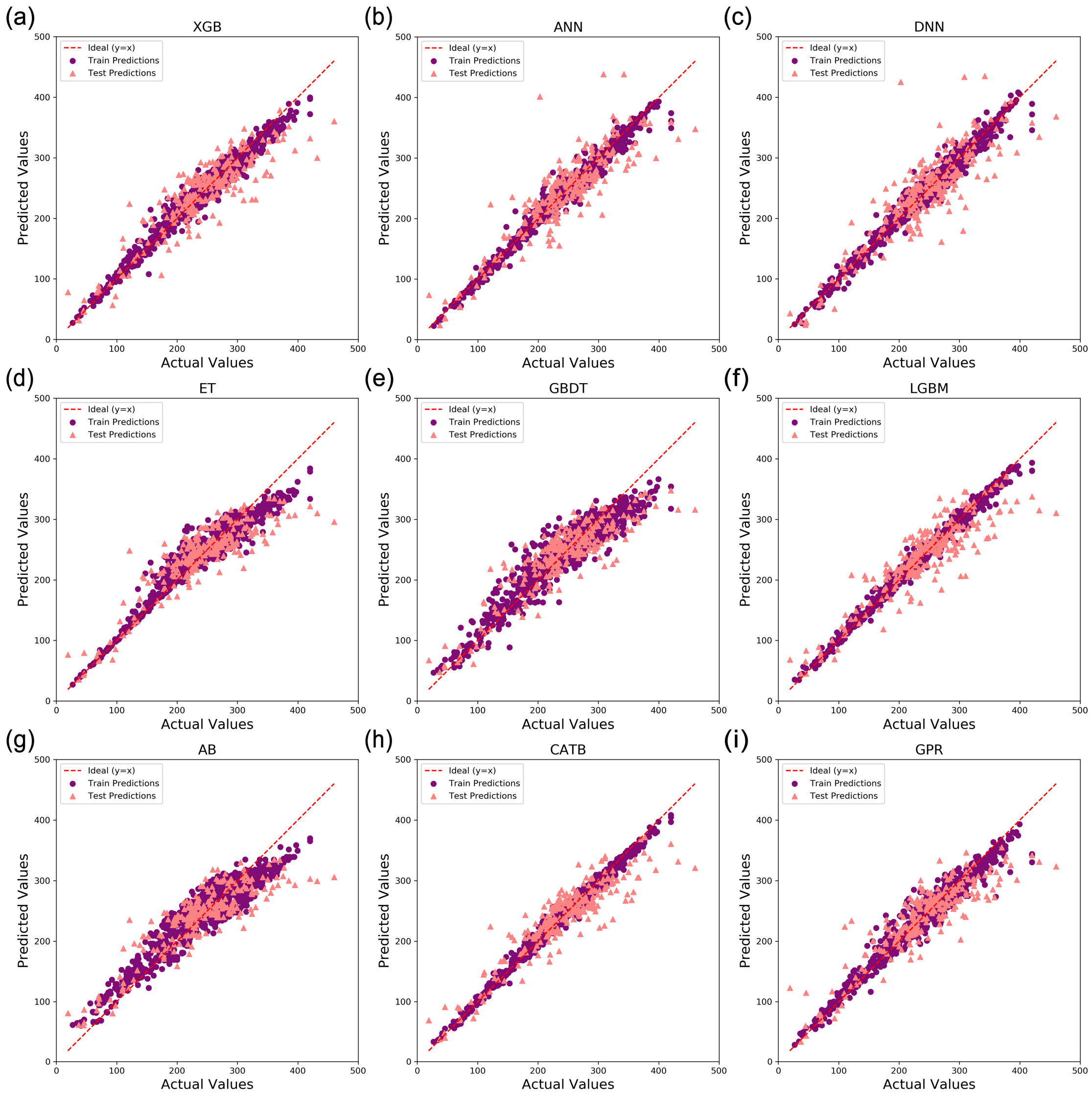

Figure S3. The prediction performance of ML models using LASSO regularization method.

Note S1. Detailed optimal parameter settings of different models.

Parameter setting of XGB

```
best_param = {
    'n_estimators': 280,
    'max_depth': 4,
    'learning_rate': 0.1,
    'min_child_weight': 1,
    'gamma': 0.1
}
XGB_model = XGBRegressor(**best_param)
XGB_model.fit(X_train, y_train)
```

Parameter setting of ANN

```
ANN_model = Sequential()
ANN_model.add(Dense (256, input_dim=X_train.shape[1], activation='relu'))
ANN_model.add(Dropout(0.2))
ANN_model.add(Dense (128, activation='relu'))
ANN_model.add(Dropout(0.1))
ANN_model.add(Dense(1, activation='linear'))
ANN_model.compile(optimizer='adam', learning_rate= 0.001, loss='mean_squared_error')
ANN_model.fit(X_train, y_train, epochs=100, batch_size=10, verbose=1))
```

Parameter setting of DNN

```
DNN_model = Sequential()
DNN_model.add(Dense (256, input_dim=X_train.shape[1], activation='relu'))
DNN_model.add(Dropout(0.2))
DNN_model.add(Dense (256, activation='relu'))
DNN_model.add(Dropout(0.1))
DNN_model.add(Dense (256, activation='relu'))
DNN_model.add(Dropout(0.1))
DNN_model.add(Dense (256, activation='relu'))
```

```
DNN_model.add(Dropout(0.1))
DNN_model.add(Dense(1, activation='relu')))
DNN_model.compile(optimizer='adam', learning_rate= 0.001, loss='mean_squared_error')
DNN_model.fit(X_train, y_train, epochs=350, batch_size=10, verbose=1))
```

Parameter setting of ET

```
best_param = {
    'n_estimators': 230,
    'max_depth': 15,
    'min_samples_split': 2,
    'min_samples_leaf': 1,
    'max_leaf_nodes': None,
    'max_samples': None
}
ET_model = ExtraTreesRegressor(**best_param)
ET_model.fit(X_train, y_train)
```

Parameter setting of GBDT

```
best_param = {
    'n_estimators': 160,
    'max_depth': 5,
    'min_samples_split': 2,
    'min_samples_leaf': 1,
    'max_leaf_nodes': None,
    'learning_rate': 0.1
}
GBDT_model = GradientBoostingRegressor(**best_param)
GBDT_model.fit(X_train, y_train)
```

Parameter setting of LGBM

```
best_param = {
    'objective': 'regression',
```

```
    ‘learning_rate’: 0.11,
    ‘n_estimators’: 100
}
LGBM_model =lgb.(**best_param)
LGBM_model.fit(X_train, y_train)
```

Parameter setting of AB

```
best_param = {
     'base_estimator': {DecisionTreeRegressor,
     'max_depth': 10,
     'min_samples_split': 2,
     'min_samples_leaf': 1,
     'max_leaf_nodes': None},
     'n_estimators': 300,
     'learning_rate': 1.0
}
AB_model = AdaBoostRegressor(**best_param)
AB_model.fit(X_train, y_train)
```

Parameter setting of CATB

```
best_param = {
    'iterations': 600,
    'depth': 6,
    'learning_rate': 0.08,
    'loss_function': 'RMSE',
    'l2_leaf_reg': 3,
    'min_data_in_leaf': 1,
    'subsample': 0.8,
    'eval_metric': 'RMSE'
}
CATB_model = CatBoostRegressor(**best_param)
```

CATB_model.fit(X_train, y_train)

Parameter setting of GPR

```
 best_param = {
     'kernel': 'ConstantKernel (1.0, (1e-3, 1e3))*RBF(1.0, (1e-2, 1e2))+ WhiteKernel(noise_level=0.1,
noise_level_bounds=(1e-10, 1e+1))',
     'n_restarts_optimizer': 15,
     'alpha': 0.1,
     'optimizer': 'fmin_l_bfgs_b'
}
GPR_model = GaussianProcessRegressor (**best_param)
GPR_model.fit(X_train, y_train)
```
